# Preclinical Pharmacokinetics of a Triple-Combination Intravaginal Ring for HIV Prevention

**DOI:** 10.3390/pharmaceutics18070829

**Published:** 2026-07-07

**Authors:** John A. Moss, Priya Srinivasan, Irina Butkyavichene, Manjula Gunawardana, Amalia E. Castonguay, John M. Cortez, Patricia Galvan, Sofia Rivera, Jining Zhang, Chuong Dinh, Angela Holder, Dawn Little, Shanon Bachman, Kristen Kelley, Christina M. Ramirez, Philippe A. Gallay, Kathleen L. Vincent, James M. Smith, Marc M. Baum

**Affiliations:** 1 Department of Chemistry, Oak Crest Institute of Science, Monrovia, CA 91016, USA; i.butkyavichene@oak-crest.org (I.B.); m.gunawardana@oak-crest.org (M.G.); a.castonguay@oak-crest.org (A.E.C.); j.cortez@oak-crest.org (J.M.C.J.);; 2 Laboratory Branch, Division of HIV Prevention, National Center for HIV, Viral Hepatitis, Sexually Transmitted Diseases, and Tuberculosis Prevention, Centers for Disease Control and Prevention, Atlanta, GA 30329, USA; pvs6@cdc.gov (P.S.); vul9@cdc.gov (C.D.);; 3 Katmai Government Services, Anchorage, AK 99515, USA; 4 Libra Management Group, Decatur, GA 30030, USA; ihv3@cdc.gov; 5 The DESA Group, Columbia, SC 29223, USA; 6 Department of Biostatistics, UCLA Fielding School of Public Health, University of California, Los Angeles (UCLA), Los Angeles, CA 90095, USA; 7 Department of Immunology and Microbiology, The Scripps Research Institute, 10550 North Torrey Pines Road, La Jolla, CA 92037, USA; 8 Department of Obstetrics and Gynecology, University of Texas Medical Branch, Galveston, TX 77555, USA

**Keywords:** intravaginal rings, HIV prevention, vaginal drug delivery, antiretroviral drug delivery

## Abstract

**Background/Objectives:** The prevention of sexual HIV-1 acquisition in women and girls in sub-Saharan Africa remains an important public health priority. Expanding the existing biomedical product portfolio to include long-acting vaginal products, such as intravaginal rings (IVRs), is expected to appeal to end users and drive adoption. **Methods:** We formulated two different triple-combination antiretroviral IVRs, both delivering the acid salt tenofovir (TFV), disoproxil fumarate (TDF), and the free base emtricitabine (FTC), with one delivering elvitegravir (EVG) as the free acid and the other as the sodium salt. The devices were evaluated for pharmacokinetics and local safety in two established preclinical models, sheep and pig-tailed macaques. **Results:** The IVRs were safe and maintained cervicovaginal fluid (CVF) drug concentrations that were above our efficacy targets derived from prior humanized mouse studies. All three agents were uniformly distributed vaginally, as evidenced by CVF and vaginal tissue measurements. Elevated drug concentrations were observed in macaque vaginal tissue samples collected three days after IVR removal, suggesting a possible forgiveness window. TFV and FTC concentrations in rectal tissue and fluid suggested potential for dual-compartment HIV-1 protection, although the vaginal-to-rectal drug transport mechanism appeared to differ across both species. The humanized mouse vaginal HIV-1 efficacy model was used to empirically compare combination effects when TDF, FTC, and EVG were co-administered, and TDF-EVG was identified as a promising combination to be developed further for IVR delivery in parallel with the triple combination.

## 1. Introduction

Recent breakthroughs in highly effective, long-acting (2–12-month duration), systemic monotherapies for HIV-1 pre-exposure prophylaxis (PrEP) have provided end-users with much-needed enhanced product options [1,2,3,4,5]. However, significant barriers to equitable access remain, particularly in low-to-middle income countries where product availability is limited and associated costs are high. New biomedical modalities for HIV-1 PrEP will need to complement existing choices and appeal to specific subgroups most vulnerable of becoming infected. Women and girls of all ages in sub-Saharan Africa accounted for 63% of all new HIV-1 infections in 2024 [6], representing an important at-risk population. Product diversity and innovation are widely regarded as key factors in driving adoption among women and amplifying the success of HIV-1 PrEP efforts [7,8]. Vaginal (i.e., topical) products are desirable options because they are currently underrepresented in the approved portfolio and have the potential to be discreet, portable, and woman-controlled, while limiting systemic exposure to drug(s) and their metabolites [9,10,11]. Long-acting products, such as intravaginal rings (IVRs) [12], are appealing because they reduce the adherence burden associated with frequent dosing regimens [13,14,15,16,17,18,19,20], such as oral Truvada^®^ (tenofovir disoproxil fumarate combined with emtricitabine, TDF-FTC) and Descovy^®^ (tenofovir alafenamide-FTC). Two large Phase III clinical trials demonstrated that a monthly IVR delivering the non-nucleoside reverse transcriptase inhibitor dapivirine (DPV) was safe and effective at preventing HIV-1 infection [21,22]. An open-label extension study in South Africa and Uganda found the incidence of HIV-1 infections during DPV IVR use to be 62% lower than the simulated placebo rate [23]. Several clinical trials found that African adolescent girls and young women preferred the DPV IVR over oral HIV-1 PrEP [24,25]. First approved in 2021 by the Medicines Control Authority of Zimbabwe, the DPV IVR has a total of 12 approvals in Africa as of October 2025 [26], representing an important milestone in expanding HIV-1 PrEP product choices to long-acting, vaginal delivery options.

We developed an innovative IVR technology for the simultaneous delivery of multiple agents, known as the pod-IVR. The platform consists of polymer-coated, solid drug tablets, “pods,” positioned in an unmedicated ring, with delivery channels exposing a predetermined surface area of the pods to cervicovaginal fluid (CVF) [27]. Each pod acts as an independent delivery module, allowing facile tuning of release characteristics on a per-pod basis by varying the polymer coating and delivery channel properties. We have used the pod-IVR platform in numerous studies, including several clinical trials involving the concurrent delivery of up to three antiretroviral (ARV) agents [28,29]. The pod-IVR platform has the advantage of enabling rapid development and in vivo evaluation of a wide range of prototypes, including the controlled delivery of salts [30,31,32,33], biologics [34,35], and live probiotic bacteria [36], which is challenging to achieve with other IVR technologies. Moreover, we demonstrated that a pod-IVR delivering TDF and FTC completely protected normally cycling pig-tailed macaques against SHIV162p3 infection in a rigorous, repeat low-dose, vaginal challenge model [37].

Here, we developed pod-IVRs for the independent delivery of TDF (fumarate salt), FTC (free-base), and elvitegravir (EVG, free-acid and sodium salt) in macaque- and human-sized IVRs. These agents have a history of use in HIV-1 PrEP, and the sodium salt of EVG is designed to increase aqueous solubility and hence the release rate. The safety, pharmacokinetics, and efficacy of the devices are discussed.

## 2. Materials and Methods

### 2.1. Materials

Tenofovir disoproxil fumarate (TDF) and emtricitabine (FTC) were purchased from Macleods Pharmaceuticals, Ltd. (Mumbai, India). Elvitegravir (EVG) was kindly provided by Gilead Sciences, Inc. (Foster City, CA, USA). Poly(vinyl alcohol) (PVA) was United States Pharmacopeia (USP) grade and obtained from Spectrum Chemical Mfg. Corp. (Gardena, CA, USA). The following stable, isotope-labeled compounds were used as internal standards for sample bioanalysis: tenofovir-*d*_6_ (Santa Cruz Biotechnology, Inc., Dallas, TX, USA), emtricitabine-^13^C,^15^N_2_ (Santa Cruz Biotechnology, Inc.), and elvitegravir-*d*_6_ (Santa Cruz Biotechnology, Inc.). Maraviroc (MVC) was used as an internal standard for the analysis of TDF and POC-TFV. Isotopically (deuterium) labeled internal standards for TFV, FTC (Moravek, Inc., Brea, CA, USA), and EVG (Toronto Research Chemicals, Inc., Toronto, ON, Canada) were used for biopsy specimen analysis.

For formulation into IVRs, the sodium salt of EVG [EVG(Na)] was prepared as follows. A methanolic sodium hydroxide solution (1.0 M) was prepared by diluting an aqueous solution (25% *w*/*v*, 6.25 M, 1.6 mL) to 10 mL with methanol. EVG (2.00 g, 4.47 mmol) was added to ethyl acetate (100 mL) in a 200 mL beaker with magnetic stirring, forming a fine suspension in ca. 10 min. Heating to 40 °C may be required to obtain a clear solution. Methanolic NaOH (1 M, 1 eq, 4.47 mL) solution was added dropwise to the resulting suspension with continued stirring for 30 min. The copious precipitate was filtered in vacuo, washed with ethyl acetate (2 × 20 mL), followed by diethyl ether (1 × 20 mL), and dried in vacuo at 50–60 °C (>12 h) to form the title compound (2.03 g, 4.32 mmol, 97%). Salt formation was confirmed via ^1^H NMR spectroscopy in DMSO-*d*_6_ by the disappearance of the EVG peak at *δ_H_* 15.45 ppm and a shift of the EVG peak at *δ_H_* 9.12 ppm to 9.07 ppm in the Na salt.

### 2.2. Fabrication of Combination Pod-Intravaginal Rings

Human- [27] and macaque-sized [38] polydimethylsiloxane (PDMS) (silicone) pod-IVRs were created according to a multistep process that was described previously [27,39,40]. Briefly, for each pod, the drug powder was blended with a lubricant (sodium stearyl fumarate, 0.5% *w*/*w*). Each powder blend was compacted into cores of 3.2 mm outer diameter in a manual tablet press using standard B-type tooling. For TDF and FTC pods used in sheep studies, an RD-10A press (Natoli Engineering, St. Charles, MO, USA) was used, and for all others, a MTCM-I press (Globe Pharma, New Brunswick, NJ, USA) was used. Drug cores of TDF and FTC for IVRs used in sheep were PVA-coated in a VFC-LAB microfluidized bed coater (Freund-Vector Corp., Marion, IA, USA) using an aqueous PVA solution (2% *w*/*v*). Drug cores of TDF and FTC for macaque IVRs and all EVG and EVG(Na) cores were dip-coated in an aqueous PVA solution (5% *w*/*v*) and dried at 60 °C for 1 h. Pods were placed in the corresponding IVR cavities and sealed in place by backfilling with room-temperature-cured silicone. Each pod (10 for IVRs sized for humans and 6 for IVRs sized for use in macaques) was matched with the appropriate configuration of mechanically punched delivery channels (Table 1).

### 2.3. In Vivo Safety and Pharmacokinetic Studies

**Figure 1:**
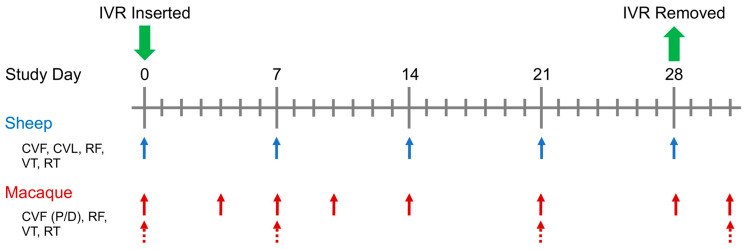
Study designs for PK studies of pod-IVRs in sheep and macaques; CVF, cervicovaginal fluid; CVL, cervicovaginal lavage; RF, rectal fluid; VT, vaginal tissue; RT, rectal tissue. Green arrows, IVR placement and removal; blue arrows, sample collection in sheep; CVF, CVL, RF, VT, RT: D0, 7, 14, 21, 28; red solid arrows, sample collection in macaques; CVF (proximal and distal to IVR), RF: D0 (baseline), 4, 7, 10, 14, 21, 28, 31; red dashed arrows VT, RT; D0 (baseline), 7, 21, 31.

Triple-combination IVRs were evaluated in nonclinical safety and pharmacokinetic (PK) studies in Merino cross-bred sheep (*N* = 3 per group) at the University of Texas Medical Branch at Galveston and pig-tailed macaques (*Macaca nemestrina*, *N* = 4 per group) at the Centers for Disease Control and Prevention. The studies were conducted under approved institutional Animal Care and Use Committee protocols and standard guidelines according to the Guide for the Care and Use of Laboratory Animals [41]. This activity was reviewed by the CDC, deemed research not involving human subjects, and was conducted consistent with applicable federal laws and CDC policies. The study design and the corresponding biological sample collection timelines, using previously published protocols [30,31,39,40,42], are shown in Figure 1. A washout period of two weeks or longer was used between IVR groups, TDF-FTC-EVG (free acid) and TDF-FTC-EVG(Na) (sodium salt).

Vaginal pH was measured in sheep and macaques prior to IVR placement and during IVR use (Figure S1). In sheep studies, additional biopsies were collected at the timepoints shown in Figure 1 and placed in formalin, followed by paraffin-embedding, sectioning, hematoxylin and eosin (H&E) staining, and review by a board-certified pathologist. Colposcopy and optical coherence tomography (OCT) imaging [43,44] were performed weekly (study days 0, 7, 14, 21, and 28) for local safety assessment of the vaginal mucosa.

### 2.4. Residual Drug Analysis in Used Intravaginal Rings

Used IVRs were returned to the Oak Crest Institute where they were analyzed for residual drug content using published methods [28,30,31,33,39,40].

Pods were excised from the used IVRs and dissolved in the appropriate media (FTC and Na-EVG, 50% *v*/*v* acetonitrile in water; TDF and EVG, acetonitrile). Aliquots (1 mL) were syringe-filtered and diluted tenfold with the appropriate media (see above). The concentrations of TDF, TFV, FTC, and EVG in the extracts were determined using an Agilent 1100 Series HPLC with diode-array (DAD) detection (Agilent Technologies, Santa Clara, CA, USA). Separation was carried out on an Atlantis T3 column (2.1 × 100 mm, 5 µm pore size; Water, Milford, MA, USA) controlled at 30 °C, using a 10 µL injection volume. The following gradient program was used (A: 25 mM H_3_PO_4_ in water; B: acetonitrile; 0.75 mL min^−1^): 1.0 min 100% A; 1.5 min ramp from 100:0 A:B to 65:35 A:B; 1.0 min ramp from 65:35 A:B to 55:45 A:B; 1.0 min ramp from 55:45 A:B to 40:60 A:B; 1.0 min ramp from 40:60 A:B to 20:80 A:B; 3.5 min hold at 20:80 A:B; 1 min ramp from 20:80 A:B to 100:0 A:B; and 2 min hold at 100:0 A:B (total run time 12 min). Elution of analytes was measured at 260 and 280 nm and the retention times were: TFV, 0.78 min; FTC, 4.44 min; POC-TFV, 4.78 min; TDF, 5.60 min; and EVG, 8.2 min.

### 2.5. Bioanalytical Methods (Oak Crest Institute of Science)

Cervicovaginal fluid samples were analyzed at the Oak Crest Institute of Science and prepared for bioanalysis as follows. Frozen (−80 °C) Dacron swabs were transferred to microcentrifuge tubes (1.5 mL), and the internal standard cocktail (1 mL; MVC, 1 µg mL^−1^; EVG-*d*_6_, 1 µg mL^−1^; TFV-*d*_6_, 10 µg mL^−1^; FTC-^13^C,^15^N_2_, 10 µg mL^−1^; dissolved in 50% *v*/*v* methanol containing 0.15% *v*/*v* trichloroacetic acid) was added. The fluid was extracted via vortex agitation for 5 min, followed by centrifugation at 12,000× *g*, 4 °C, for 10 min. Supernatant aliquots (700 µL) were transferred to 96-well collection plates (2 mL), along with calibration and quality control standards, and evaporated using a concentrator system (Savant SC210A Plus, Thermo Fisher Scientific, Inc., Hudson, NH, USA). The dry samples were reconstituted in high-purity water (700 µL) and processed using Impact Protein Precipitation Plates (Phenomenex, Torrance, CA, USA) according to the manufacturer’s instructions. The samples were redried and reconstituted in mobile phase (100 µL) for analysis via liquid chromatography–tandem mass spectrometry (LC-MS/MS). A similar approach was used for the bioanalysis of CVL samples, except that the initial swab extraction step was unnecessary.

Three different bioanalytical methods were used to analyze the CVF and CVL samples. All three methods involved LC-MS/MS analysis using an Agilent 1100 Series HPLC system interfaced to an API 3000 triple-quadrupole tandem mass spectrometer (AB Sciex, Framingham, MA, USA) with a Turbo Ion Spray electrospray ionization (ESI) source. The following parameters were used throughout: 5 µL injection volume, 0.8 mL min^−1^ flow rate, and column temperature maintained at 40 °C.

Separation of TDF, POC-TFV, and TFV was carried out on an Agilent XDB-C18 column (2.1 × 50 mm, 3.5 µm particle size) equipped with an Agilent C8 guard column (2.1 × 12.5 mm, 5 µm particle size). The following gradient program was used (A: 0.1% *v*/*v* formic acid in water; B: 0.1% *v*/*v* formic acid in acetonitrile): 0.25 min 100% A; 1.25 min ramp from 100:0 A:B to 70:30 A:B; 1.0 min ramp from 70:30 A:B to 50:50 A:B; 1.0 min hold at 50:50 A:B; 1.5 min ramp from 50:50 A:B to 95:5 A:B; and 0.5 min ramp from 95:5 A:B to 100:0 A:B (total run time 5.5 min). The analyte retention times were: TFV, 0.27 min; POC-TFV, 1.83 min; MVC, 2.38 min; and TDF, 2.60 min. The measured transition ions, *m*/*z*, in positive ESI mode were as follows: for TFV, 288.1 atomic mass units (amu) (parent) and 176.2 amu (product); for POC-TFV, 404.1 amu (parent) and 176.1 amu (product); for tenofovir disoproxil, 520.0 amu (parent) and 270.0 amu (product); for MVC (IS), 514.7 amu (parent) and 280.6 amu (product); and for TFV-*d*_6_ (IS), 293.1 amu (parent) and 181.2 amu (product).

Separation of FTC was carried out on a Waters Atlantis T3 column (2.1 × 50 mm, 5 µm particle size) equipped with a Waters Atlantis T3 guard column (2.1 × 10 mm, 5 µm particle size). The following gradient program was used (A: 0.1% *v*/*v* formic acid in water; B: 0.1% *v*/*v* formic acid in acetonitrile): 0.25 min 100% A; 1.25 min ramp from 100:0 A:B to 70:30 A:B; 1.0 min ramp from 70:30 A:B to 50:50 A:B; 1.0 min hold at 50:50 A:B; 1.5 min ramp from 50:50 A:B to 95:5 A:B; 0.5 min ramp from 95:5 A:B to 100:0 A:B; and 1.0 min hold at 100:0 A:B (total run time 6.5 min). The FTC retention time was 1.63 min. The measured transition ions, *m*/*z*, in positive ESI mode were as follows: for FTC, 248.0 atomic mass units (amu) (parent) and 130.0 amu (product); and for FTC-^13^C,^15^N_2_ (IS), 251 amu (parent) and 133 amu (product).

Separation of EVG was carried out on an Agilent XDB-C18 column (2.1 × 50 mm, 3.5 µm particle size) equipped with an Agilent C8 guard column (2.1 × 12.5 mm, 5 µm particle size). The following gradient program was used (A: 0.1% *v*/*v* formic acid in water; B: 0.1% *v*/*v* formic acid in acetonitrile): 3 min ramp from 60:40 A:B to 0:100 A:B; 1.0 min ramp from 0:100 A:B to 60:40 A:B; and 1.0 min hold at 60:40 A:B (total run time 5.0 min). The EVG retention time was 1.77 min. The measured transition ions, *m*/*z*, in positive ESI mode were as follows: for EVG, 448.5 atomic mass units (amu) (parent) and 344.2 amu (product); and for EVG-*d*_6_ (IS), 454.2 amu (parent) and 350.2 amu (product).

Bioanalytical methods were qualified and run in accordance with FDA guidelines [45]. Assay lower limits of quantification (LLOQs/LOQs) are presented in the Supplementary Materials (Table S1).

The naïve lavage fluid for CVL sample collection in sheep contained exogenous LiCl (10 mM). The undiluted vaginal fluid volume collected during the CVL procedure was measured by analyzing the reduction in Li^+^ signal (i.e., dilution by collected CVF) via ion chromatography (IC), according to methods discussed in detail elsewhere [29,33,46].

### 2.6. Bioanalytical Methods (Centers for Disease Control and Prevention)

The bioanalysis of vaginal and rectal tissues and rectal fluid samples was performed at the Centers for Disease Control and Prevention. Concentrations of TFV, FTC, and EVG were measured using LC-MS/MS (Shimadzu Scientific, Columbus, MD; Sciex, Foster City, CA, USA). Rectal swab and tissue biopsy specimens were extracted with methanol (80% *v*/*v*, 500 µL) containing deuterium-labeled internal standards for TFV, FTC, and EVG. Swab samples were centrifuged at 13,600 rcf for 5 min to remove particulates, and biopsy specimens were sonicated for 30 min followed by centrifugation at 13,600 rcf for 5 min. Aliquots (350 µL) of each supernatant were transferred to a microtiter plate, evaporated to near dryness, and reconstituted in mobile phase A (0.2% *v*/*v* formic acid in water, 150 µL). A 10 µL injection was loaded onto a UK-C18 column (100 × 1 mm; Imtakt, Portland, OR, USA). Analytes were separated using a linear gradient of mobile phase B (0.2% *v*/*v* formic acid in acetonitrile) from 2% to 98% over 5 min. Mass spectrometric detection was performed in positive electrospray ionization mode using multiple reaction monitoring (MRM). The monitored transitions, *m*/*z*, were: TFV, 288→176.3 and 288→159.1; FTC, 248.1→130.1 and 248.1→113.1; and EVG, 448.2→344.1 and 448.2→143.1. Quantification was performed using Analyst software (Sciex, version 1.7.3.) based on calibration curves ranging from 0.5 to 2000 ng mL^−1^, prepared in plasma for biopsy samples and in aqueous solution for swab samples.

TFV-DP and FTC-TP were measured in vaginal and rectal biopsies as described previously [47]. Methanol (80% *v*/*v*, 500 µL) was added to the specimen followed by the internal standard. Analyte concentrations were measured using an automated online weak anion-exchange solid-phase extraction method coupled with ion-pair chromatography–MS/MS [48].

Bioanalytical methods were qualified and run in accordance with FDA guidelines [45]. Assay lower limits of quantification (LLOQs/LOQs) are presented in the Supplementary Materials (Table S1).

### 2.7. Humanized Mouse Efficacy Studies

In vivo efficacy studies using bone marrow/liver/thymus (BLT) humanized (hu) mice were carried out at the Department of Animal Resources (DAR), The Scripps Research Institute, animal biosafety level 3 facilities, under protocols approved by the Institutional Animal Care and Use Committee at The Scripps Research Institute (Permit Number: 13-0001). The study protocols adhered strictly to the recommendations in the Guide for the Care and Use of Laboratory Animals of the National Institutes of Health. In an effort to minimize suffering, surgeries were performed using sodium pentobarbital anesthesia, and animals were euthanized via cervical dislocation. The study is reported in accordance with Animal Research: Reporting of In Vivo Experiments (ARRIVE) guidelines.

BLT hu-mice were generated according to previously described methods [49,50,51,52,53,54]. Vaginal drug dosing of BLT hu-mice with TDF-EVG and FTC-EVG in phosphate-buffered saline followed by a single, atraumatic vaginal HIV-1 challenge and subsequent analysis of HIV-1 infection were carried out using protocols described in detail elsewhere [49,50,51,54,55,56,57,58,59,60,61].

### 2.8. Statistical and Data Analysis

All data processing, visualization, and statistical analyses were performed using GraphPad Prism version 10.6.1 (GraphPad Software, Inc., La Jolla, CA, USA) and RStudio 2025.09.1. Unless otherwise stated, data are summarized using medians with interquartile ranges (IQRs) for concentration data and means ± standard deviation (*SD*) for graphical time-course displays. All statistical tests were two-sided, and statistical significance was assessed at *α* = 0.05.

Values below the assay LOQ were treated as left-censored. Sample concentrations at timepoints with the IVR in place that were below the corresponding lower limit of quantification (LOQ) of the assay (*C_LLQ_*) were imputed according to Equation (1) [29]:

(1) $$C_{LLQ}=\frac{\mathrm{LOQ}}{2\times \tilde{m}}$$

where *C_LLQ_* is the analyte LOQ in concentration units, LOQ is the assay limit of quantification on a per sample basis, and $\tilde{m}$ is the median sample mass (for swab-collected fluid and tissue biopsies) or the median sample volume (for lavage-derived samples after dilution correction), consistent with prior work. Samples/timepoints with a high proportion of below the limit of quantitation (BLQ) values (as specified in the corresponding tables) were not summarized for that analyte–matrix combination. The proportion of quantifiable samples (% > LOQ) is reported for each analyte and matrix.

Pre-specified comparisons were conducted as follows:

- In vivo release rates, estimated from the residual drug mass remaining in used IVRs, were compared between IVR formulations (EVG free acid versus EVG sodium salt) within species using an unpaired *t*-test with Welch’s correction.
- Within-animal paired comparisons of drug concentrations across sampling locations were performed using the Wilcoxon matched-pairs signed-rank test, including: (a) sheep CVF versus dilution-corrected CVL collected at the same visit; (b) macaque CVF proximal versus distal to the IVR collected at the same timepoint; and (c) macaque vaginal tissue biopsies collected proximal versus distal to the IVR at the same timepoint.
- Concentration ratios between anatomic compartments were computed within animal and timepoint (paired ratios), and are presented descriptively using box plots (median, IQR, and range).

Dose–response relationships and drug–drug interaction analyses in humanized mouse studies were performed using the median-effect (Chou–Talalay) method [62,63] implemented in CompuSyn [64]. Combination effects were quantified using the combination index (*CI*; *CI* < 1 synergism, *CI* = 1 additivity, *CI* > 1 antagonism) and dose-reduction index (DRI). Fraction affected (*F_a_*) values of 0.0025 and 0.99 were used to represent 0% and 100% efficacy, respectively, for model fitting and inference.

## 3. Results

### 3.1. Intravaginal Ring Configurations and In Vivo Release Rates

The four IVR configurations evaluated here are summarized in Table 1. All IVR configurations consisted of triple ARV combinations based on the TDF-FTC backbone with the addition of either EVG free acid (EVG) or the sodium salt [EVG(Na)]. Human-sized sheep IVRs typically contained between 2.5 and 4 times more of each active pharmaceutical ingredient (API) than the corresponding IVR sized for a macaque.

**Figure 2:**
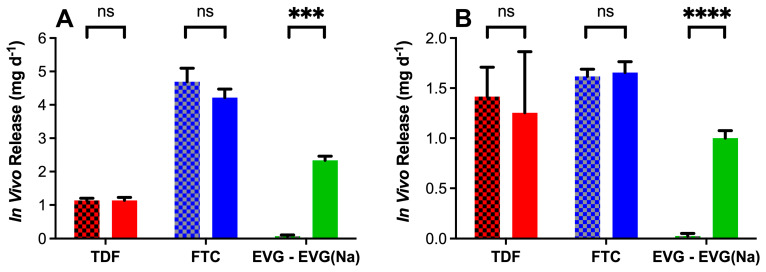
Comparison of in vivo release of TDF, FTC, and EVG from IVRs delivering TDF-FTC-EVG (checkered bars) and TDF-FTC-EVG(Na) (shaded bars) in (**A**) sheep and (**B**) macaques. Comparisons: ns, difference not significant (*p* > 0.05); ***, *p* ≤ 0.001; ****, *p* ≤ 0.0001.

The in vivo drug release rates for all IVR configurations calculated based on the residual drug in the used devices are listed in Table 1 and compared in Figure 2. An unpaired, two-tailed *t*-test (parametric) with Welch’s correction was used to compare in vivo drug release rates between TDF-FTC-EVG and TDF-FTC-EVG(Na) groups in sheep (Figure 2A) and macaques (Figure 2B). The release rates of TDF and FTC were not significantly different in these groups: TDF, sheep, *p* > 0.9999; TDF, macaques, *p* = 0.6589; FTC, sheep, *p* = 0.1748; FTC, macaques, *p* = 0.5878. However, the release rates of EVG and EVG(Na) were significantly different: sheep, *p* = 0.0006; macaques, *p* < 0.0001.

### 3.2. Drug Product Local Safety

No adverse events or unusual abnormalities related to the test articles were observed during the course of the studies. The sheep and macaques remained healthy and maintained appropriate appetite and body weight, based on intermittent physical examinations and twice-daily cage-side observations. The animals did not exhibit any signs of discomfort and there were no significant findings on any measure of safety that would suggest toxicity. No systematic changes in vaginal pH were observed with the IVRs in place (Figure S1). No IVR expulsions were observed.

In sheep, colposcopic assessment of the vaginal mucosa was conducted prior to the placement of IVRs and during IVR use on days 0, 7, 14, 21 and 28 for each IVR. Colposcopy findings were normal (Tables S2 and S3) throughout the 28-day study. Neither the TDF-FTC-EVG or the TDF-FTC-EVG(Na) IVR formulation led to toxicity, including irritation, epithelial disruption, or ulcerations, as evidenced by colposcopy. Observations showed mostly pink color, intact vessels, and no epithelial disruption.

Assessment of the vaginal epithelium via optical coherence tomography (OCT) was conducted at baseline and during use of each IVR on days 0, 7, 14, 21 and 28. Fluctuations in epithelial thickness corresponding to 1–2 cell layers in the epithelium were observed (Figure S2) and were not a local safety concern.

Sheep vaginal and rectal biopsies were collected on study days 0, 7 (except P8, TDF-FTC-EVG IVR), and 28. The specimens were H&E stained, sectioned, and examined by a board-certified pathologist. Most of the slides did not show any pathologic changes during microscopic assessment. A few sections showed minimal inflammatory infiltrates, mostly either in the epithelial or submucosal layer. Three sections showed mild changes consistent with an inflammatory response, with mild multifocal infiltrates in the submucosa and lamina propria and small foci of hemorrhage. The observations described above are consistent with a very limited inflammatory infiltrate involving the epithelium, lamina propria, and submucosa of the normal vagina.

### 3.3. Summary of PK Measurements in Sheep and Macaques

Antiretroviral drug and drug metabolite concentrations in key anatomic compartments for HIV-1 PrEP are summarized for sheep (Table 2 and Table 3) and macaques (Table 4 and Table 5) with triple-combination IVRs. Samples that were below the limit of quantification (LOQ) of the assay were imputed according to Equation (1) in Section 2.8. The amount of cervicovaginal fluid (CVF) collected via swab sampling was measured as mass (mg), while lavage (CVL) was measured as volume (mL), after compensation for dilution [46]. Molar concentrations assumed a matrix density of 1 g cm^−3^.

Median TFV_total_ and FTC concentrations in CVF were similar across both IVR groups and across animal models (macaques and sheep). Concentrations of EVG were ca. 100–200× higher in sheep IVR formulations containing the sodium salt (Table 2 and Table 3), while they were only ca. 10× higher in the corresponding macaque IVRs (Table 4 and Table 5). However, the EVG concentrations in CVF were ca. 200× higher in macaques versus sheep for the TDF-FTC-EVG IVR group.

### 3.4. Drug Metabolite Mole Fractions

In the phosphonate ester prodrug TDF, the two free phosphonic acid moieties of the TFV backbone are protected as methylene(*iso*propyl)carbonate (i.e., isoproxil, POC) groups. In vivo enzymatic metabolism hydrolyzes the esters to the mono-isoproxil TFV intermediate (POC-TFV) and then to TFV. The mean POC-TFV mole fraction—*χ*(*POC-TFV*) = [POC-TFV]/{[TDF] + [POC-TFV} + [TFV]}, on a molar basis—in paired CVF measurements was consistent within each species and across IVR types (Table 6). However, its metabolism was more extensive in sheep, and *χ*(*POC-TFV*) was ca. 8 times lower in sheep than in macaques (Table 6). The TDF concentrations in CVF and CVL were BLQ in most sheep samples (Table 2 and Table 3), and mean *χ*(*TDF*) values in macaque CVF ranged between 2.8% and 6.4%.

The mean *χ*(*TFV-DP*) in tissue specimens is indicative of how extensively the TFV reservoir is phosphorylated to TFV-DP, the metabolite responsible for inhibiting HIV-1 reverse transcriptase. Overall, the measured *χ*(*TFV-DP*) values were low, ranging between 0.12 and 2.2% across the different tissue types in both species (Table 6). In sheep, the mean *χ*(*TFV-DP*) in rectal tissue was ca. double that in the corresponding vaginal tissue samples (Table 6). The *χ*(*TFV-DP*) in macaque vaginal tissue was typically 10× higher than that in sheep vaginal tissue.

### 3.5. Cervicovaginal Fluid Drug Concentration-Time Profiles

**Figure 3:**
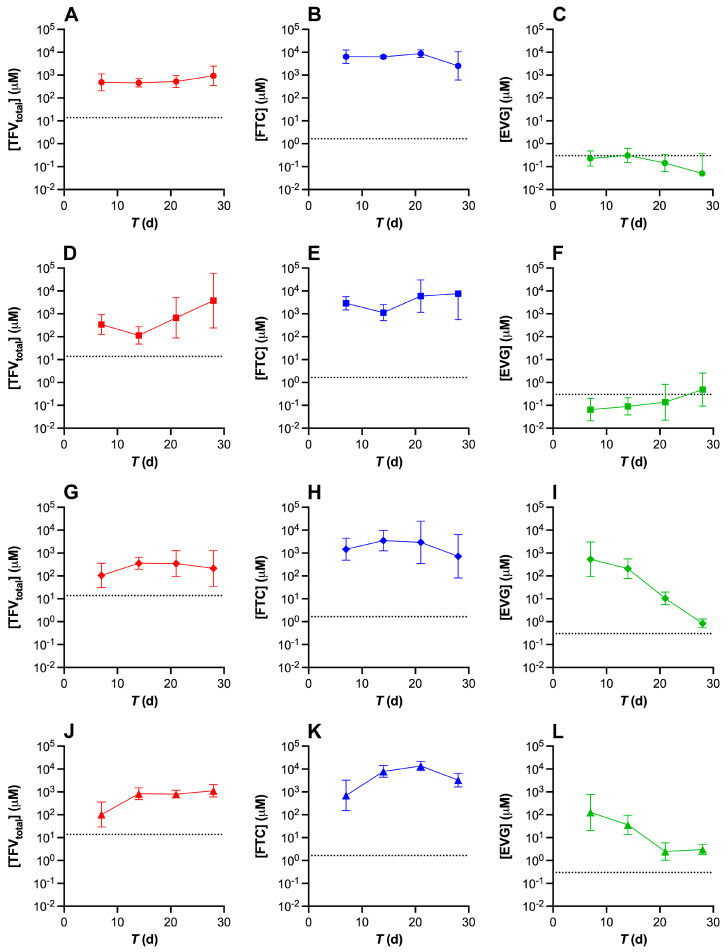
Cervicovaginal fluid concentration–time profiles for drugs delivered from pod-IVRs in sheep (mean ± *SD*). Sample key: red, TFV_total_ (1 µM = 0.29 µg g^−1^); blue, FTC (1 µM = 0.25 µg g^−1^); green, EVG (1 µM = 0.45 µg g^−1^). Circles (**A**–**C**), TDF-FTC-EVG IVR, CVF; squares (**D**–**F**), TDF-FTC-EVG IVR, CVL (corrected for dilution); diamonds (**G**–**I**), TDF-FTC-EVG(Na) IVR, CVF; triangles (**J**–**L**), TDF-FTC-EVG(Na) IVR, CVL (corrected for dilution). Broken horizontal lines represent targets associated with 100% protection in humanized mice [61]: TFV, 14 µM; FTC, 1.7 µM; EVG, 0.30 µM.

**Figure 4:**
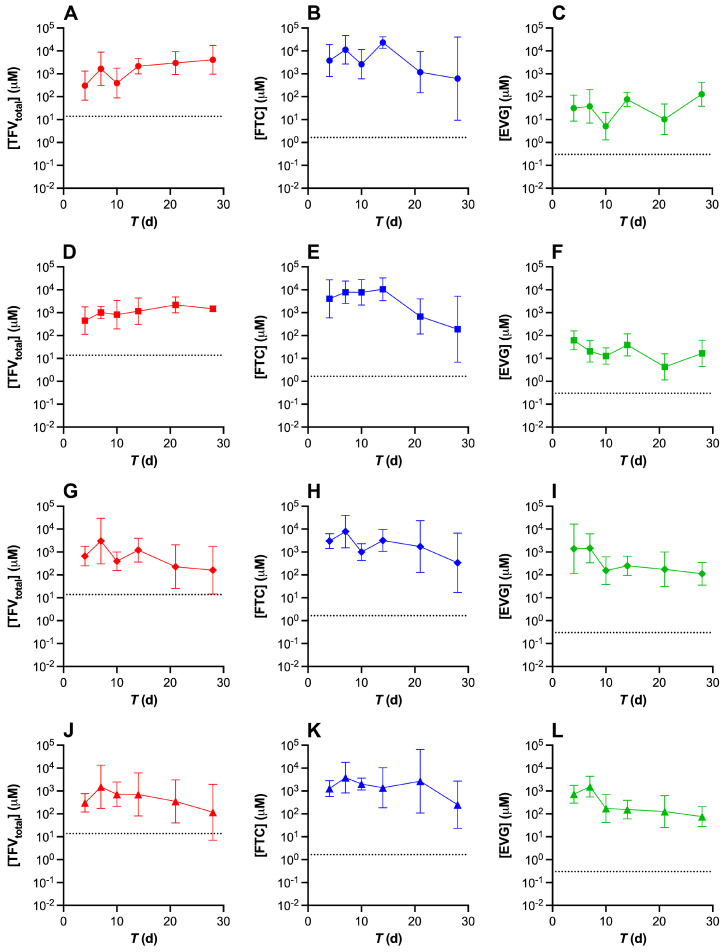
Cervicovaginal fluid concentration–time profiles for drugs delivered from pod-IVRs in macaques (mean ± *SD*). Sample key: red, TFV_total_ (1 µM = 0.29 µg g^−1^); blue, FTC (1 µM = 0.25 µg g^−1^); green, EVG (1 µM = 0.45 µg g^−1^). Circles (**A**–**C**), TDF-FTC-EVG IVR, proximal to IVR; squares (**D**–**F**), TDF-FTC-EVG IVR, distal to IVR; diamonds (**G**–**I**), TDF-FTC-EVG(Na) IVR, proximal to IVR; triangles (**J**–**L**), TDF-FTC-EVG(Na) IVR, distal to IVR. Broken horizontal lines represent targets associated with 100% protection in humanized mice [61]: TFV, 14 µM; FTC, 1.7 µM; EVG, 0.30 µM.

The CVF drug concentration–time profiles for sheep and macaques are shown in Figure 3 and Figure 4, respectively. For ease of comparison across datasets, all concentrations are expressed in µM and the same *y*-axis range was used for all plots. Concentration–time plots for TDF and its metabolites, POC-TFV and TFV, are included in the Supplementary Materials (Figures S3 and S4). Importantly, the drug concentrations were well above the 100% efficacious targets (dotted horizontal lines, Figure 3 and Figure 4) established in previous humanized mouse studies using vaginal TDF-FTC-EVG dosing [61]. The only exception was EVG in sheep using the TDF-FTC-EVG IVRs (Figure 3C,F).

Paired drug concentrations in CVF and CVL (corrected for dilution) sheep samples were compared using a nonparametric, Wilcoxon matched-pairs signed rank test. The following pairs were not significantly different (*p* > 0.05): TDF-FTC-EVG IVR group, TFV_total_ (*p* = 0.9097); FTC (*p* = 0.6772); EVG (*p* = 0.7334); TDF-FTC-EVG(Na) IVR group, FTC (*p* = 0.1099). Only two concentration pairs were significantly different: TDF-FTC-EVG(Na) IVR group, TFV_total_ (*p* = 0.0068); EVG (*p* = 0.0093).

Paired drug concentrations in proximal and distal CVF macaque samples were compared using a nonparametric, Wilcoxon matched-pairs signed rank test. All drug concentration pairs were not significantly different (*p* > 0.05): TDF-FTC-EVG IVR, TFV_total_ (*p* = 0.2292); FTC (*p* = 0.6231); EVG (*p* = 0.4389); TDF-FTC-EVG(Na) IVR, TFV_total_ (*p* = 0.3165); FTC (*p* = 0.3165); EVG (*p* = 0.5457).

### 3.6. Vaginal Tissue Drug Concentration-Time Profiles

**Figure 5:**
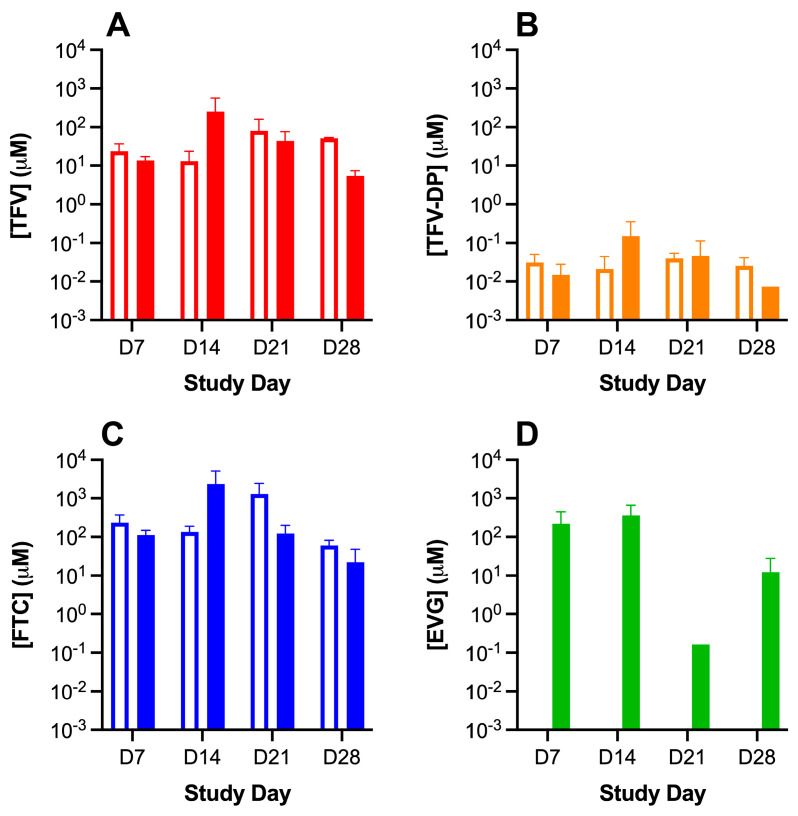
Vaginal tissue drug concentrations (mean ± *SD*) in sheep receiving triple-combination pod-IVRs. (**A**) TFV (1 µM = 0.29 µg g^−1^); (**B**) TFV-DP (1 µM = 10^−6^ fmol g^−1^); (**C**) FTC (1 µM = 0.25 µg g^−1^); (**D**) EVG (1 µM = 0.45 µg g^−1^). Sample key: open bars, TDF-FTC-EVG IVRs; shaded bars, TDF-FTC-EVG(Na) IVRs. All EVG samples in the TDF-FTC-EVG IVR group were BLQ and omitted from panel (**D**). All FTC-TP measurements were BLQ and omitted (Table 2 and Table 3).

**Figure 6:**
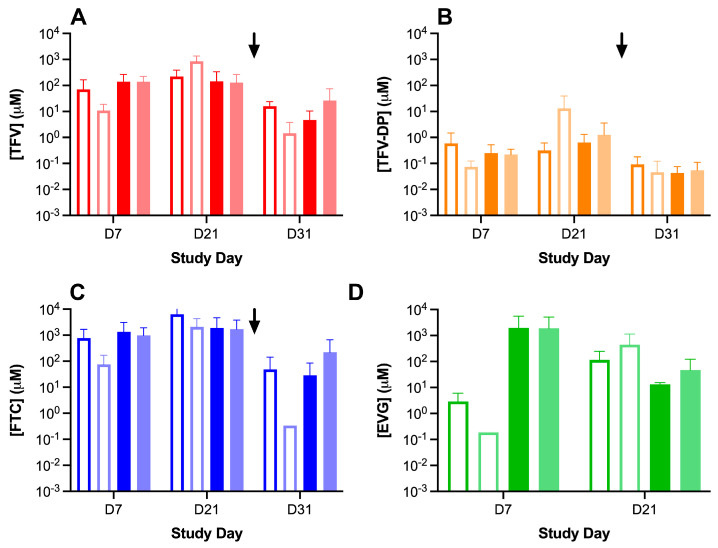
Vaginal tissue drug concentrations (mean ± *SD*) in macaques receiving triple-combination pod-IVRs. The IVRs were removed on study day 28 (arrows). (**A**) TFV (1 µM = 0.29 µg g^−1^); (**B**) TFV-DP (1 µM = 10^−6^ fmol g^−1^); (**C**) FTC (1 µM = 0.25 µg g^−1^); (**D**) EVG (1 µM = 0.45 µg g^−1^). Sample key: open bars, TDF-FTC-EVG IVRs; shaded bars, TDF-FTC-EVG(Na) IVRs; dark colors, biopsies collected proximal to the IVRs; pale colors, samples collected distal to the IVRs. Note clipped error bar for D21, TDF-FTC-EVG IVR, proximal in panel (**C**). All EVG samples on Day 31 were BLQ and were omitted from panel (**D**). Most FTC-TP measurements were BLQ and were omitted (Table 4 and Table 5).

Drug concentrations in vaginal biopsy samples—sheep (Figure 5) and macaques (Figure 6)—are presented in micromolar units and with the same *y*-axis range to facilitate comparison across measurements. Vaginal tissue TFV concentrations were similar across both animal models and IVR types, while TFV-DP metabolite concentrations were considerably higher in macaques. The D31 macaque TFV-DP vaginal tissue concentrations, three days after IVR removal, were comparable to corresponding measurements in sheep with the IVRs in place. Macaque vaginal tissue EVG concentrations also were higher than those in sheep prior to removing the IVRs.

Paired drug concentrations in proximal and distal VT macaque samples were compared using a nonparametric, Wilcoxon matched-pairs signed-rank test. All drug concentration pairs were not significantly different (*p* > 0.05): TDF-FTC-EVG IVR, TFV (*p* = 0.6875); TFV-DP (*p* = 0.9453); FTC (*p* = 0.1484); TDF-FTC-EVG(Na) IVR, TFV (*p* = 0.8438); TFV-DP (*p* = 0.6875); FTC (*p* = 0.4609); EVG (*p* = 0.9453). EVG concentrations in the TDF-FTC-EVG IVR group were omitted from the analysis due to the large number of BLQ measurements (Table 4 and Table 5).

### 3.7. Rectal Tissue and Fluid Drug Concentration-Time Profiles

**Figure 7:**
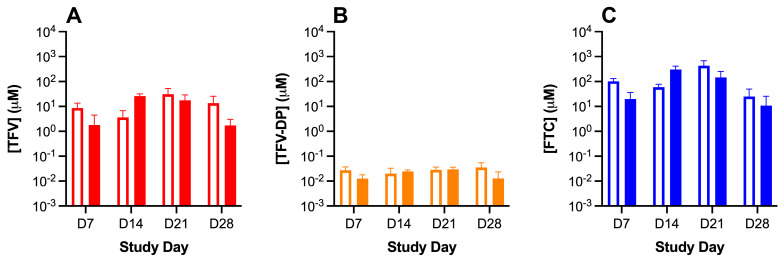
Rectal tissue drug concentrations (mean ± *SD*) in sheep receiving triple-combination pod-IVRs. (**A**) TFV (1 µM = 0.29 µg g^−1^); (**B**) TFV-DP (1 µM = 10^−6^ fmol g^−1^); (**C**) FTC (1 µM = 0.25 µg g^−1^). Sample key: open bars, TDF-FTC-EVG IVRs; shaded bars, TDF-FTC-EVG(Na) IVRs. The majority of EVG concentration measurements were BLQ. All FTC-TP measurements were BLQ and omitted (Table 2 and Table 3).

Rectal tissue drug concentrations in sheep specimens are shown in Figure 7 but were BLQ in most corresponding macaque samples (Table 4 and Table 5).

**Figure 8:**
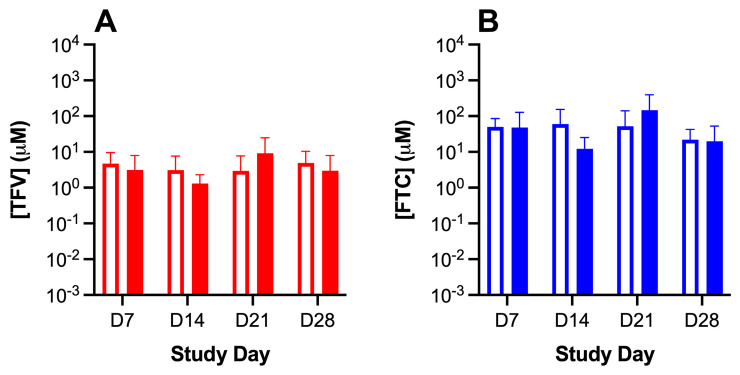
Rectal fluid drug concentrations (mean ± *SD*) in sheep receiving triple-combination pod-IVRs. (**A**) TFV (1 µM = 0.29 µg g^−1^); (**B**) FTC (1 µM = 0.25 µg g^−1^). Sample key: open bars, TDF-FTC-EVG IVRs; shaded bars, TDF-FTC-EVG(Na) IVRs. All EVG concentration measurements were BLQ and omitted (Table 2 and Table 3).

**Figure 9:**
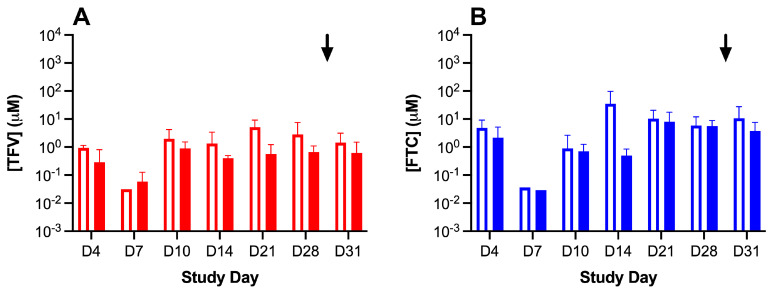
Rectal fluid drug concentrations (mean ± *SD*) in macaques receiving triple-combination pod-IVRs. The IVRs were removed on study day 28 (arrows). (**A**) TFV (1 µM = 0.29 µg g^−1^); (**B**) FTC (1 µM = 0.25 µg g^−1^). Sample key: open bars, TDF-FTC-EVG IVRs; shaded bars, TDF-FTC-EVG(Na) IVRs. All EVG concentration measurements were BLQ and omitted (Table 4 and Table 5).

Rectal fluid drug concentrations in sheep (Figure 8) and macaques (Figure 9) were only quantifiable for TFV and FTC, not EVG. They were generally higher for FTC and in sheep (Table 2, Table 3, Table 4 and Table 5), but similar for both IVR types.

### 3.8. Analyte Distribution Across Anatomic Compartments

**Figure 10:**
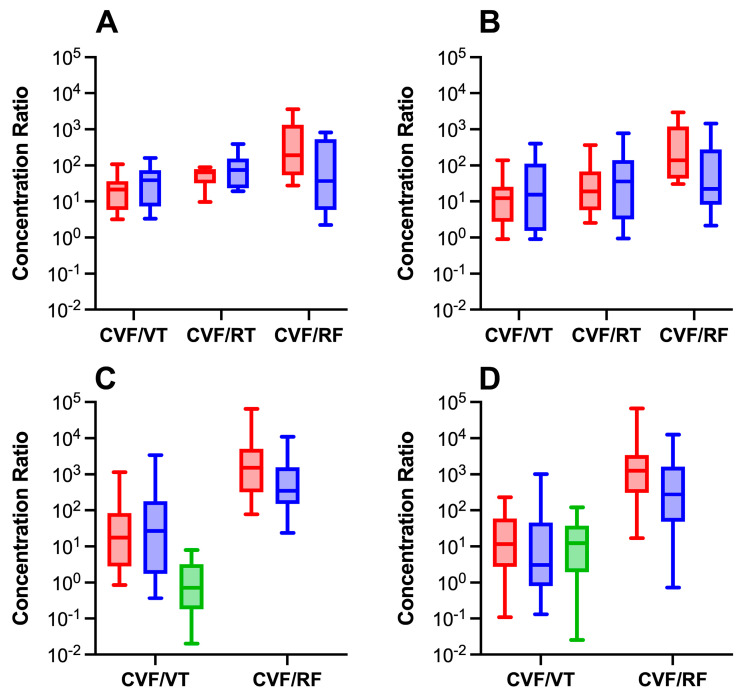
Box plots of paired concentration ratios in sheep (**A**,**B**) and macaques (**C**,**D**) with the IVRs in place. The box extends from the 25th to 75th percentiles, with the horizontal line in the box representing the median; whiskers represent the lowest and highest datum. (**A**,**C**) TDF-FTC-EVG IVRs; (**B**,**D**) TDF-FTC-EVG(Na) IVRs; red, TFV; blue, FTC; green, EVG. Sheep EVG samples were not included due to the high number of BLQ measurements.

Paired concentration ratios (i.e., for the same animal and timepoint) across anatomic compartments provide insights on how the drugs are partitioning following IVR delivery into the cervicovaginal fluid (CVF). The corresponding ratios are presented below for sheep (Figure 10A,B) and macaques (Figure 10C,D).

Concentration gradients were higher in macaque samples than in sheep samples, particularly between the vaginal and rectal compartments.

### 3.9. Vaginal HIV-1 Prevention Efficacy of Drug Combinations in Humanized Mice

The median-effect (Chou–Talalay) model based on mass action [62,63] unifies fundamental biochemical and biophysical equations to enable quantitative pharmacodynamic analysis of dose-effect relationships in complex biological systems. It provides an unbiased view of drug interactions and combination effects without requiring a preexisting knowledge of underlying mechanisms.

We applied the median-effect model to analyze data from vaginal HIV-1 prevention studies in bone marrow/liver/thymus (BLT) humanized (hu) mice dosed topically with TDF, FTC, TDF-FTC, and TDF-FTC-EVG [54,60,61]. We observed a slight antagonism between the nucleotide reverse transcriptase inhibitors (NRTIs) TDF and FTC (i.e., two-drug combination) [54], which was amplified to acute antagonism by the addition of EVG in the triple-drug combination [61]. Here, we sought to determine if this antagonism could be alleviated by either omitting TDF or FTC from the three-drug combination.

**Figure 11:**
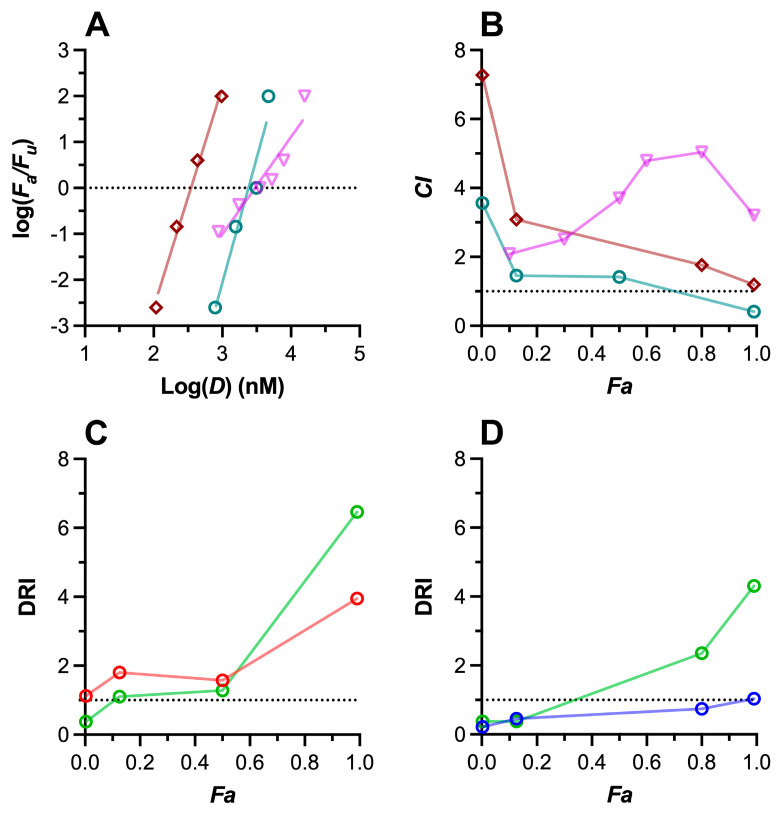
Analysis of efficacy in vaginal HIV-1 prevention using BLT hu-mice (*N* = 8–10 per dosing group) using the median-effect model and dosing with dual-drug combinations consisting of TDF-EVG and FTC-EVG compared with the triple combination of TDF-FTC-EVG reported previously [61]. *F_a_*, fraction affected; *F_u_*, fraction unaffected; *D*, dose (nM). (**A**) Log-log dose–response relationships derived from TDF-EVG (teal, open circles), FTC-EVG (dark red, open diamonds), and the triple combination (magenta, open triangles). (**B**) Combination index (*CI*) plot comparing TDF-EVG (teal, open circles), FTC-EVG (dark red, open diamonds), and TDF-FTC-EVG (magenta, open triangles). *CI* > 1 antagonism; *CI* = 1 (broken line), additive effect; *CI* < 1 synergism. Dose-reduction index (DRI) plots for TDF-EVG (**C**) and FTC-EVG (**D**). The DRI of 1 shown as a broken line represents no dose reduction relative to the drugs evaluated individually. Red, TDF; blue, FTC, green; EVG.

The median-effect plots for the TDF-EVG and FTC-EVG combinations followed the mass action principle (TDF-EVG, *R*^2^ = 0.946; FTC-EVG, *R*^2^ = 0.992, Figure 11A). Drug combination effects as a function of HIV-1 prevention efficacy (*F_a_*) were assessed quantitatively using combination index (*CI*) plots [62,63]. If the effect of the drug combination is additive, *CI* = 1. Antagonism is defined by *CI* > 1 and synergism by *CI* < 1. The *CI* values at various *F_a_* levels are presented for TDF-EVG and FTC-EVG (Figure 11B), overlaid with the corresponding plots from the TDF-FTC-EVG triple combination reported previously [61]. As they approached 100% efficacy in HIV-1 prevention (*F_a_* = 1), the FTC-EVG combination was essentially additive (*CI* = 1.2), while the TDF-EVG combination was synergistic (*CI* = 0.41), in sharp contrast to the antagonism observed for the triple combination of TDF-FTC-EVG (*CI* = 3.2) [61].

The median-effect model also was used to calculate the dose-reduction index (DRI) over the range of *F_a_* values (Figure 11C,D). The DRI represents the number of times the dose of each drug in the combination can be reduced (synergism) or needs to be increased (antagonism) at a given *F_a_* relative to the individual drug doses [62,63]. In the TDF-EVG combination, high DRI values (TDF > 3.0; EVG > 4.4, Figure 11C) were observed for *F_a_* > 0.8 (i.e., in the target efficacy range). The corresponding DRI values for each agent were lower for the FTC-EVG combination (FTC > 0.75; EVG > 2.4, Figure 11C).

## 4. Discussion

We developed pod-IVRs for the independent, controlled delivery of TDF (fumarate salt), FTC (free-base), and EVG (free-acid and sodium salt) in macaque- and human-sized IVRs. The rationale for drug selection was based on the hypothesis that the vaginal HIV-1 PrEP efficacy of the dual-nucleotide reverse transcriptase inhibitor (NRTI) backbone of TDF and FTC would be augmented when combined with another ARV agent from a different mechanistic class. Oral Truvada^®^ (TDF-FTC) is an approved regimen for HIV-1 PrEP. We previously showed that a pod-IVR delivering TDF and FTC was safe in a first in-human clinical trial [29] and conferred full protection from SHIV162p3 infection in a rigorous, repeat low-dose, vaginal challenge model using normally cycling female pig-tailed macaques [37]. For the third agent, we selected the integrase strand transfer inhibitor (INSTI) EVG, based in part on the successful systemic HIV-1 prevention monotherapy in women of another INSTI, cabotegravir [2]. EVG also provides added antiviral activity against transmitted HIV-1 variants that have reduced susceptibility to TFV or FTC.

The pod-IVRs delivered TDF and FTC at high rates (>1 mg d^−1^) in sheep and pig-tailed macaques (Table 1) and did not lead to any safety concerns related to product use. As expected, the release of the water-insoluble agent EVG was greatly accelerated by formulating the compound as its sodium salt: 31× in sheep and 50× in macaques. There were no drug–drug interactions resulting from the different EVG forms in the IVRs. The release rate of EVG, or its salt, could be controlled without altering the TDF or FTC release rates. While the sample sizes used in both animal models were small, they are typical for large mammal preclinical PK and safety studies, especially in the exploratory phase of product development. Drug and drug metabolite PK differences were observed across both species that cannot be attributed exclusively to differences in IVR release rates. It is unclear which species will be better at predicting drug PK in humans.

Elevated CVF drug and drug metabolite concentration plateaus were maintained for TFV and FTC during IVR use but were several orders of magnitude lower for EVG in the TDF-FTC-EVG IVR groups (Table 2, Table 3, Table 4 and Table 5). The EVG CVF concentration–time profiles in the TDF-FTC-EVG(Na) IVR groups were species-dependent. In sheep, a steep concentration decrease was observed over 28 days of use (Figure 3I,L), while in macaques the corresponding CVF EVG concentrations were higher and stable over time following a ca. 10-fold decrease over the first 10 days of use (Figure 3L and Figure 4I). The differences are not related to drug depletion from the IVRs but are likely associated with gradual pH-buffering by CVF. The pH of sheep CVF was considerably lower than that of macaque CVF (Figure S1), suggesting more efficient generation of EVG free acid in the IVR pods. This hypothesis is supported by the observation that the sheep CVF EVG concentrations on day 28, just prior to IVR removal, were similar across the two formulations (i.e., EVG free-acid and sodium salt, Figure 3I,L) suggesting that most of the pod-contained API had reverted to EVG, from Na-EVG, over the 28 days. Drug concentration gradients in CVF were not observed in both species. In sheep, most paired drug concentrations in CVF and CVL, corrected for dilution using lithium as an exogenous tracer [46], were not statistically different. In macaques, paired drug concentrations in CVF samples collected proximally and distally to the IVRs also matched statistically. A homogenous drug distribution in the vaginal lumen is desirable for optimal product efficacy.

Vaginal tissue biopsy drug and drug metabolite concentrations followed similar trends to those observed for CVF, with the expected gradients across anatomic compartments (Figure 10). The HIV-1 PrEP efficacy of a vaginal product based on ARV drugs is generally believed to be related to the agent’s concentration and distribution in vaginal tissues. The phosphorylated NRTI metabolites, TFV-DP and FTC-TP, are the active moieties against HIV-1. The metabolite FTC-TP was not detected in most samples, possibly due to species-dependent intracellular phosphorylation efficiency differences and/or the lower analytical sensitivity relative to TFV-DP (Table S1). The TFV-DP tissue concentrations in macaques were similar to those in our clinical trial [29], while the concentrations were lower in sheep (Table 2, Table 3, Table 4 and Table 5, Figure 5 and Figure 6). Vaginal tissue EVG concentrations could only be quantified in the Na-EVG group in sheep but were measured for both IVR groups in macaques, as expected based on the corresponding trends noted for drug concentrations in CVF. Macaque vaginal tissue concentrations for all analytes were not statistically different if collected proximal or distal relative to the IVRs, in agreement with the corresponding observations in CVF and further suggesting uniform drug distribution in the vaginal tract. Concentrations of TFV, TFV-DP and FTC in vaginal tissues remained elevated three days after IVR removal, suggesting a possible forgiveness window (i.e., increased duration of HIV-1 protection) if the IVR is removed before vaginal intercourse. These findings need to be substantiated with additional PK studies, preferably in women.

Ideally, an IVR for HIV-1 PrEP would protect both vaginal and rectal anatomic compartments from sexual transmission. Receptive anal intercourse (RAI) leads to a higher risk of HIV-1 infection per sex act and, for women, there is the possibility of both vaginal and rectal exposure during a single sex act [65,66,67]. In a previous pod-IVR clinical trial that included a TDF-FTC group, we measured unexpectedly high rectal fluid drug concentrations, along with drug–drug interactions resulting from the inclusion of the inhibitor/antagonist of chemokine receptor CCR5 maraviroc in the combination [28]. We observed consistent TFV, TFV-DP, and FTC exposure in sheep rectal tissue samples, but not in macaques (Table 2, Table 3, Table 4 and Table 5, Figure 7). However, rectal fluid TFV and FTC concentrations were quantifiable for both species, but were ca. 10× higher for sheep. There was no appreciable drop in macaque rectal fluid drug concentrations three days after IVR removal. The mechanism for drug transfer from the CVF to the rectal compartment appears to be species-dependent. Figure 10 shows paired concentration ratios normalized to CVF across anatomic compartments. In sheep (Figure 10A,B), these ratios gradually increase, suggesting drug diffusion from the CVF via vaginal tissues to rectal tissues and eventually to rectal fluids. These data align closely with our observations of a TDF-FTC pod-IVR in women where the CVF to RF drug concentration ratios were ca. 100 [28]. In macaques, the rectal tissue compartment is missing because the drug concentrations were not quantifiable overall, suggesting possible direct fluid transfer across the two compartments. The potential of dual-compartment HIV-1 prevention from IVR use needs to be explored in future PK-pharmacodynamic studies and cannot be inferred purely from the data presented here.

We previously used the BLT hu-mouse model to empirically study the effect of drug combinations on vaginal HIV-1 PrEP using the Chou–Talalay model [62,63,68], based on the median-effect principle of the mass action law [54,60,61]. While TDF-FTC exhibited mild antagonism [54], the TDF-FTC-EVG combination was acutely antagonistic [61]. That study allowed us to identify CVF drug concentrations in the triple combination that were associated with 100% protection, and these were used as efficacy targets in the current report. Figure 3 and Figure 4 show that both pod-IVR types either meet or exceed these targets, with the exception of the EVG concentrations in the TDF-FTC-EVG IVR group in sheep for some timepoints. Preliminary data in the previous report [61] suggested that omitting TDF from the triple combination significantly reduced the observed antagonism. We explored the combination effect of TDF-EVG and FTC-EVG in more detail here (Figure 11). The TDF-EVG combination led to a greater DRI than FTC-EVG (Figure 11C,D) for both drugs. Both double combinations were able to overcome the acute antagonism of the triple combination, with the TDF-EVG combination exhibiting mild synergy at high efficacy values (Figure 11B). It is important to note that the triple-combination pod-IVRs in the current study were able to safely overcome the antagonism observed in hu-mice. However, there are some limitations to extrapolating results from these efficacy studies based on a bolus vaginal drug dose to continuous IVR dosing. The pharmacokinetic complementarity of TFV-DP and FTC-TP, in terms of terminal half-lives in vaginal tissues, may warrant co-administration to maximize the forgiveness period after the IVR is removed, as discussed by Gallay et al. [61].

If future studies support the premise that the TDF-FTC backbone is not needed for an effective HIV-1 PrEP IVR, an optimal candidate product would be based on either dual-drug combination discussed above. Reformulation of the TDF-FTC-EVG/EVG(Na) pod-IVR to eliminate one of the NRTI agents simply would entail omitting the corresponding drug pods and would have no impact on the release characteristics of the remaining drugs. Doncel and colleagues at CONRAD are developing an on-demand, fast-dissolving insert delivering TAF and EVG for vaginal and rectal HIV-1 PrEP and post-exposure prophylaxis (PEP), and these efforts have proceeded to clinical trials [69,70,71,72]. A TDF-EVG(Na) IVR would constitute a logical complement to this promising product. The next round of IVR formulation will need to provide additional pH buffering of the Na-EVG reservoir, as women with lactobacillus-rich vaginal microbiomes have a uniquely acidic vaginal pH (typically 3.8) [73] that is far more acidic than the vaginal pH of non-human mammals, including sheep and macaques [74].

## 5. Conclusions

Pod-intravaginal rings were formulated to deliver TDF-FTC in combination with EVG (as the free-acid or sodium salt) in sheep and macaques. The IVRs were safe and successfully maintained vaginal fluid drug concentrations above our efficacy targets.

## Figures and Tables

**Table 1:** Intravaginal ring configurations and in vivo release rates.

Configuration	API	Delivery Channel Surface Area (mm^2^)	Drug Mass (mg)	In Vivo Release Rate (mg d^−1^)
IVR-1 ^1^	TDF	7.1	186	1.14 ± 0.06
	FTC	1.8	145	4.69 ± 0.40
	EVG	1.8	114	0.075 ± 0.033
IVR-2 ^1^	TDF	7.1	186	1.14 ± 0.09
	FTC	1.8	144	4.22 ± 0.25
	EVG(Na)	1.8	114	2.34 ± 0.13
IVR-3 ^2^	TDF	5.3	47	1.42 ± 0.29
	FTC	0.88	47	1.62 ± 0.07
	EVG	3.5	45	0.02 ± 0.02
IVR-4 ^2^	TDF	5.3	47	1.26 ± 0.61
	FTC	0.88	48	1.66 ± 0.11
	EVG(Na)	3.5	45	1.00 ± 0.08

^1^ Sheep. ^2^ Macaque.

**Table 2:** Summary of ARV drug and drug metabolite concentrations in sheep with TDF-FTC-EVG IVRs in place (days 7–28).

Analyte, Matrix	*n* ^1^	% > LLQ ^2^	Median (IQR) ^3^	
TDF, CVF	12	50	3.05 × 10^−3^ (1.20 × 10^−3^–9.05 × 10^−3^) μg g^−1^	4.86 × 10^−3^ (1.96 × 10^−3^–14.2 × 10^−3^) μM
POC-TFV, CVF	12	100	6.92 (1.38–10.7) μg g^−1^	17.2 (3.41–26.5) μM
TFV, CVF	12	100	167 (82.5–232) μg g^−1^	583 (287–809) μM
TFV_total_, ^4^ CVF			170 (86.6–242) μg g^−1^	591 (301–842) μM
FTC, CVF	12	100	1.69 × 10^3^ (1.18 × 10^3^–2.26 × 10^3^) μg g^−1^	6.84 × 10^3^ (4.76 × 10^3^–9.12 × 10^3^) μM
EVG, CVF	12	100	80.1 × 10^−3^ (43.2 × 10^−3^–163 × 10^−3^) μg g^−1^	179 × 10^−3^ (96.3 × 10^−3^–363 × 10^−3^) μM
TDF, CVL ^5^	12	8.3	n.a. ^6^	n.a. ^6^
POC-TFV, CVL ^5^	12	100	6.00 (0.972–27.3) μg mL^−1^	14.9 (2.41–67.6) μM
TFV, CVL ^5^	12	100	69.7 (42.2–672) μg mL^−1^	243 (147–2.34 × 10^3^) μM
TFV_total_, ^4^ CVL ^5^	12		72.9 (44.9–715) μg mL^−1^	254 (156–2.49 × 10^3^) μM
FTC, CVL ^5^	12	100	714 (320–2.71 × 10^3^) μg mL^−1^	2.89 × 10^3^ (1.30 × 10^3^–11.0 × 10^3^) μM
EVG, CVL ^5^	12	100	42.3 × 10^−3^ (22.4 × 10^−3^–113 × 10^−3^) μg mL^−1^	94.4 × 10^−3^ (50.2 × 10^−3^–253 × 10^−3^) μM
TFV, VT	12	92	9.01 (2.68–15.2) μg g^−1^	31.4 (9.34–52.8) μM
TFV-DP, VT	12	67	32.0 × 10^−3^ (7.35 × 10^−3^–41.6 × 10^−3^) fmol g^−1^	32.0 × 10^−3^ (7.35 × 10^−3^–41.6 × 10^−3^) μM
FTC, VT	12	92	34.9 (17.7–60.6) μg g^−1^	141 (71.7–245) μM
FTC-TP, VT	12	0	n.a. ^6^	n.a. ^6^
EVG, VT	12	8.3	n.a. ^6^	n.a. ^6^
TFV, RT	12	83	3.02 (1.14–5.25) μg g^−1^	10.5 (3.97–18.3) μM
TFV-DP, RT	12	92	27.6 × 10^−3^ (20.2 × 10^−3^–35.8 × 10^−3^) fmol g^−1^	27.6 (20.2 × 10^−3^–35.8 × 10^−3^) μM
FTC, RT	12	92	17.5 (11.4–34.9) μg g^−1^	70.7 (46.0–141) μM
FTC-TP, RT	12	0	n.a. ^6^	n.a. ^6^
EVG, RT	12	33	n.a. ^6^	n.a. ^6^
TFV, RF	12	83	0.552 (0.134–2.40)	1.92 (0.467–8.36)
FTC. RF	12	92	4.72 (0.789–13.6)	19.1 (3.19–55.2)
EVG. RF	12	0	n.a. ^6^	n.a. ^6^

^1^ Number of samples analyzed; ^2^ Proportion of analyzed samples with quantifiable analyte concentrations; ^3^ Interquartile range (25th to 75th percentile); ^4^ Total TFV (i.e., TDF + POC-TFV + TFV) on a molar basis; ^5^ Corrected for dilution during the CVL procedure; ^6^ Not included in the analysis due to high proportion of BLQ samples. CVF, cervicovaginal fluid; CVL, cervicovaginal lavage; VT, vaginal tissue; RT, rectal tissue; RF, rectal fluid.

**Table 3:** Summary of ARV drug and drug metabolite concentrations in sheep with TDF-FTC-EVG(Na) IVRs in place (days 7–28).

Analyte, Matrix	*n* ^1^	% > LLQ ^2^	Median (IQR) ^3^	
TDF, CVF	12	33	n.a. ^6^	n.a. ^6^
POC-TFV, CVF	12	100	0.875 (61.0 × 10^−3^–14.1) μg g^−1^	2.17 (0.151–34.9) μM
TFV, CVF	12	100	70.9 (42.3–159) μg g^−1^	247 (147–554) μM
TFV_total_, ^4^ CVF			95.3 (42.5–160) μg g^−1^	332 (148–557) μM
FTC, CVF	12	100	676 (225–1.56 × 10^3^) μg g^−1^	2.73 × 10^3^ (911–6.30 × 10^3^) μM
EVG, CVF	12	100	18.4 (1.85–160) μg g^−1^	41.2 (4.14–356) μM
TDF, CVL ^5^	12	8.3	n.a. ^6^	n.a. ^6^
POC-TFV, CVL ^5^	12	100	8.31 (3.54–28.7) μg mL^−1^	20.6 (8.78–71.1) μM
TFV, CVL ^5^	12	100	204 (105–310) μg mL^−1^	711 (365–1.08 × 10^3^) μM
TFV_total_, ^4^ CVL ^5^			211 (118–325) μg mL^−1^	735 (412–1.13 × 10^3^) μM
FTC, CVL ^5^	12	100	1.35 × 10^3^ (656–2.55 × 10^3^) μg mL^−1^	5.45 × 10^3^ (2.65 × 10^3^–10.3 × 10^3^) μM
EVG, CVL ^5^	12	100	4.45 (1.13–23.8) μg mL^−1^	9.94 (2.51–53.1) μM
TFV, VT	12	100	5.03 (2.69–11.2) μg g^−1^	17.5 (9.38–39.1) μM
TFV-DP, VT	12	42	7.35 × 10^−3^ (7.35 × 10^−3^–33.5 × 10^−3^) fmol g^−1^	7.35 × 10^−3^ (7.35 × 10^−3^–33.5 × 10^−3^) μM
FTC, VT	12	100	26.0 (15.2–60.2) μg g^−1^	105 (61.7–243) μM
FTC-TP, VT	12	0	n.a. ^6^	n.a. ^6^
EVG, VT	12	67	11.4 (73.5 × 10^−3^–93.2) μg g^−1^	25.5 (0.164–208) μM
TFV, RT	12	75	2.15 (0.536–6.23) μg g^−1^	7.49 (1.87–21.7) μM
TFV-DP, RT	12	75	22.4 × 10^−3^ (12.8 × 10^−3^–25.6 × 10^−3^) fmolg g^−1^	22.4 × 10^−3^ (12.8 × 10^−3^–25.6 × 10^−3^) μM
FTC, RT	12	92	13.0 (2.85–50.6) μg g^−1^	52.5 (11.5–205) μM
FTC-TP, RT	12	0	n.a. ^6^	n.a. ^6^
EVG, RT	12	50	1.49 (66.7 × 10^−3^–10.4) μg g^−1^	3.32 (0.149–23.3) μM
TFV, RF	12	83	0.113 (54.1 × 10^−3^–1.05) μg g^−1^	0.394 (0.188–3.67) μM
FTC. RF	12	92	0.644 (0.180–8.48) μg g^−1^	2.61 (0.729–34.3) μM
EVG. RF	12	0	n.a. ^6^	n.a. ^6^

^1^ Number of samples analyzed; ^2^ Proportion of analyzed samples with quantifiable analyte concentrations; ^3^ Interquartile range (25th to 75th percentile); ^4^ Total TFV (i.e., TDF + POC-TFV + TFV) on a molar basis; ^5^ Corrected for dilution during the CVL procedure; ^6^ Not included in the analysis due to high proportion of BLQ samples. CVF, cervicovaginal fluid; CVL, cervicovaginal lavage; VT, vaginal tissue; RT, rectal tissue; RF, rectal fluid.

**Table 4:** Summary of ARV drug and drug metabolite concentrations in macaques with TDF-FTC-EVG IVRs in place (days 4–28).

Analyte, Matrix	*n* ^1^	% > LLQ ^2^	Median (IQR) ^3^	
TDF, CVF, proximal ^4^	24	63	8.49 (0.150–24.2) μg g^−1^	13.4 (0.236–38.1) μM
POC-TFV, CVF, proximal ^4^	24	100	286 (105–751) μg g^−1^	710 (260–1.86 × 10^3^) μM
TFV, CVF, proximal ^4^	24	100	157 (55.8–507) μg g^−1^	546 (194–1.76 × 10^3^) μM
TFV_total_, ^5^ CVF, proximal^4^			395 (191–1.06 × 10^3^) μg g^−1^	1.38 × 10^3^ (665–3.70 × 10^3^) μM
FTC, CVF, proximal ^4^	24	100	1.85 × 10^3^ (329–3.90 × 10^3^) μg g^−1^	7.47 × 10^3^ (1.33 × 10^3^–15.8 × 10^3^) μM
EVG, CVF, proximal ^4^	24	100	12.2 (4.37–42.8) μg g^−1^	27.1 (9.76–95.5) μM
TDF, CVF, distal ^6^	24	63	3.99 (0.214–41.9) μg g^−1^	6.27 (0.337–65.9) μM
POC-TFV, CVF, distal ^6^	24	100	219 (163–474) μg g^−1^	543 (405–1.18 × 10^3^) μM
TFV, CVF, distal ^6^	24	100	111 (69.3–230) μg g^−1^	388 (241–801) μM
TFV_total_, ^5^ CVF, distal ^6^			323 (193–593) μg g^−1^	1.12 × 10^3^ (672–2.06 × 10^3^) μM
FTC, CVF, distal ^6^	24	100	1.25 × 10^3^ (337–2.68 × 10^3^) μg g^−1^	5.07 × 10^3^ (1.37 × 10^3^–10.8 × 10^3^) μM
EVG, CVF, distal ^6^	24	96	10.0 (3.75–21.2) μg g^−1^	22.3 (8.38–47.2) μM
TFV, VT, proximal ^4^	8	88	37.5 (3.72–65.6) μg g^−1^	131 (13.0–228) μM
TFV-DP, VT, proximal ^4^	8	100	193 × 10^3^ (89.9 × 10^3^–499 × 10^3^) fmol g^−1^	0.193 (89.9 × 10^−3^–0.499) μM
FTC, VT, proximal ^4^	8	100	176 (11.7–924) μg g^−1^	711 (47.1–3.74 × 10^3^) μM
FTC-TP, VT, proximal ^4^	8	13	n.a. ^7^	n.a. ^7^
EVG, VT, proximal ^4^	8	63	2.12 (98.0 × 10^−3^–26.6) μg g^−1^	4.73 (0.219–59.4) μM
TFV, VT, distal ^6^	8	100	50.2 (3.16–196) μg g^−1^	175 (11.0–683) μM
TFV-DP, VT, distal ^6^	8	100	177 × 10^3^ (64.2 × 10^3^–344 × 10^3^) fmol g^−1^	0.177 (64.2 × 10^−3^–0.344) μM
FTC, VT, distal ^6^	8	100	32.0 (8.15–284) μg g^−1^	129 (33.0–1.15 × 10^3^) μM
FTC-TP, VT, distal ^6^	8	25	n.a. ^7^	n.a. ^7^
EVG, VT, distal ^6^	8	50	0.664 (83.3 × 10^−3^–39.2) μg g^−1^	1.48 (0.186–87.6) μM
TFV, RT	8	0	n.a. ^7^	n.a. ^7^
TFV-DP, RT	8	38	n.a. ^7^	n.a. ^7^
FTC, RT	8	0	n.a. ^7^	n.a. ^7^
FTC-TP, RT	8	38	n.a. ^7^	n.a. ^7^
EVG, RT	8	0	n.a. ^7^	n.a. ^7^
TFV, RF	24	79	0.149 (30.0 × 10^−3^–1.12) μg g^−1^	0.520 (0.104–3.90) μM
FTC. RF	24	71	0.475 (9.00 × 10^−3^–2.12) μg g^−1^	1.92 (36.4 × 10^−3^–8.58) μM
EVG. RF	24	0	n.a. ^7^	n.a. ^7^

^1^ Number of samples analyzed; ^2^ Proportion of analyzed samples with quantifiable analyte concentrations; ^3^ Interquartile range (25th to 75th percentile); ^4^ Sample collected proximal to IVR; ^5^ total TFV (i.e., TDF + POC-TFV + TFV) on a molar basis; ^6^ Sample collected distal to IVR; ^7^ Not included in the analysis due to high proportion of BLQ samples. CVF, cervicovaginal fluid; VT, vaginal tissue; RT, rectal tissue; RF, rectal fluid.

**Table 5:** Summary of ARV drug and drug metabolite concentrations in macaques with TDF-FTC-EVG(Na) IVRs in place (days 7–28).

Analyte, Matrix	*n* ^1^	% > LLQ ^2^	Median (IQR) ^3^	
TDF, CVF, proximal ^4^	24	75	6.68 (0.198–34.4) μg g^−1^	10.5 (0.311–54.2) μM
POC-TFV, CVF, proximal ^4^	24	92	126 (52.7–280) μg g^−1^	312 (131–694) μM
TFV, CVF, proximal ^4^	24	100	54.7 (31.9–207) μg g^−1^	190 (111–722) μM
TFV_total_, ^5^ CVF, proximal ^4^			181 (82.5–418) μg g^−1^	630 (287–1.46 × 10^3^) μM
FTC, CVF, proximal ^4^	24	100	617 (253–1.40 × 10^3^) μg g^−1^	2.49 × 10^3^ (1.02 × 10^3^–5.64 × 10^3^) μM
EVG, CVF, proximal ^4^	24	100	82.4 (66.8–432) μg g^−1^	184 (149–964) μM
TDF, CVF, distal ^6^	24	79	6.89 (1.26–26.1) μg g^−1^	10.8 (1.98–41.0) μM
POC-TFV, CVF, distal ^6^	24	92	126 (15.9–298) μg g^−1^	312 (39.4–739) μM
TFV, CVF, distal ^6^	24	96	45.2 (15.6–152) μg g^−1^	157 (54.4–528) μM
TFV_total_, ^5^ CVF, distal ^6^			140 (62.1–413) μg g^−1^	487 (216–1.44 × 10^3^) μM
FTC, CVF, distal ^6^	24	100	543 (108–933) μg g^−1^	2.20 × 10^3^ (437–3.77 × 10^3^) μM
EVG, CVF, distal ^6^	24	100	117 (32.3–364) μg g^−1^	261 (72.2–812) μM
TFV, VT, proximal ^4^	8	100	19.6 (12.5–56.3) μg g^−1^	68.1 (43.4–196) μM
TFV-DP, VT, proximal ^4^	8	88	209 × 10^3^ (72.5 × 10^3^–687 × 10^3^) fmol g^−1^	0.208 (72.5 × 10^−3^–0.687) μM
FTC, VT, proximal ^4^	8	100	180 (77.8–409) μg g^−1^	726 (314–1.66 × 10^3^) μM
FTC-TP, VT, proximal ^4^	8	25	n.a. ^7^	n.a. ^7^
EVG, VT, proximal ^4^	8	100	33.4 (6.37–82.7) μg g^−1^	74.7 (14.2–185) μM
TFV, VT, distal ^6^	8	100	30.6 (20.1–49.7) μg g^−1^	107 (70.0–173) μM
TFV-DP, VT, distal ^6^	8	100	188 × 10^3^ (86.9 × 10^3^–332 × 10^3^) fmol g^−1^	0.188 (86.9 × 10^−3^–0.332) μM
FTC, VT, distal ^6^	8	100	183 (44.5–555) μg g^−1^	738 (180–2.25 × 10^3^) μM
FTC-TP, VT, distal ^6^	8	13	n.a. ^7^	n.a. ^7^
EVG, VT, distal ^6^	8	100	46.7 (5.54–129) μg g^−1^	104 (12.4–289) μM
TFV, RT	8	13	n.a. ^7^	n.a. ^7^
TFV-DP, RT	8	50	11.0 × 10^3^ (7.46 × 10^3^–36.2 × 10^3^)	11.0 × 10^−3^ (7.46 × 10^−3^–36.2 × 10^−3^)
FTC, RT	8	25	n.a. ^7^	n.a. ^7^
FTC-TP, RT	8	13	n.a. ^7^	n.a. ^7^
EVG, RT	8	0	n.a. ^7^	n.a. ^7^
TFV, RF	24	75	99.0 × 10^−3^ (26.2 × 10^−3^–0.186) μg g^−1^	0.345 (91.3 × 10^−3^–0.647) μM
FTC. RF	24	71	0.180 (7.22 × 10^−3^–0.598) μg g^−1^	0.729 (29.2 × 10^−3^–2.42) μM
EVG. RF	24	0	n.a. ^7^	n.a. ^7^

^1^ Number of samples analyzed; ^2^ Proportion of analyzed samples with quantifiable analyte concentrations; ^3^ Interquartile range (25th to 75th percentile); ^4^ Sample collected proximal to IVR; ^5^ total TFV (i.e., TDF + POC-TFV + TFV) on a molar basis; ^6^ Sample collected distal to IVR; ^7^ Not included in the analysis due to high proportion of BLQ samples. CVF, cervicovaginal fluid; VT, vaginal tissue; RT, rectal tissue; RF, rectal fluid.

**Table 6:** Mole fractions of TDF metabolites in different anatomic compartments.

IVR Type, Animal Model	Analyte, Matrix	Mean ± *SD* (%)
TDF-FTC-EVG, Sheep	*χ*(*POC-TFV*), CVF	6.3 ± 8.4
	*χ*(*POC-TFV*), CVL	6.2 ± 8.9
	*χ*(*TFV-DP*), VT	0.14 ± 0.15
	*χ*(*TFV-DP*), RT	0.29 ± 0.17
TDF-FTC-EVG(Na), Sheep	*χ*(*POC-TFV*), CVF	6.0 ± 10.3
	*χ*(*POC-TFV*), CVL	6.1 ± 11.0
	*χ*(*TFV-DP*), VT	0.12 ± 0.07
	*χ*(*TFV-DP*), RT	0.28 ± 0.33
TDF-FTC-EVG, Macaque	*χ*(*POC-TFV*), CVF, proximal	53.0 ± 12.9
	*χ*(*POC-TFV*), CVF, distal	56.9 ± 14.1
	*χ*(*TFV-DP*), VT, proximal	2.15 ± 4.31
	*χ*(*TFV-DP*), VT, distal	1.1 ± 1.5
TDF-FTC-EVG(Na), Macaque	*χ*(*POC-TFV*), CVF, proximal	46.6 ± 20.8
	*χ*(*POC-TFV*), CVF, distal	46.5 ± 19.7
	*χ*(*TFV-DP*), VT, proximal	0.87 ± 1.2
	*χ*(*TFV-DP*), VT, distal	0.70 ± 1.4

CVF, cervicovaginal fluid; VT, vaginal tissue; RT, rectal tissue.

## Data Availability

The data presented in this study are available upon request from the corresponding authors.

## Supplementary Materials

The following supporting information can be downloaded at: https://www.mdpi.com/article/10.3390/pharmaceutics18070829/s1. Table S1. Analytical Limits of Quantitation (LOQs) and Ranges for Drug and Drug Metabolites in Sheep and Macaque Specimens. All values are reported in (ng sample^−1^) unless otherwise specified. CVF, Cervicovaginal fluid; CVL, cervicovaginal lavage; VT, vaginal tissue; RT, rectal tissue; RF, rectal fluid. Table S2. Colposcopic Findings during TDF-FTC-EVG IVR Study in Sheep (*N* = 3). Table S3. Colposcopic Findings during TDF-FTC-EVG(Na) IVR Study in Sheep (*N* = 3). Figure S1. Temporal Vaginal pH Measurements. Box plots, line at median and floating bars represent minimum to maximum values. (A) Sheep; blue, TDF-FTC-EVG IVR group; red, TDF-FTC-EVG(Na) IVR group. (B) Macaques; TDF-FTC-EVG IVR group; (C) Macaques; TDF-FTC-EVG(Na) IVR group. Figure S2. Temporal Thickness Variation of Vaginal Epithelium in Sheep Measured by Optical Coherence Tomography (OCT). (A) TDF-FTC-EVG IVR group. (B) TDF-FTC-EVG(Na) IVR group. Figure S3. Concentration-time Plots for TDF and its Metabolites in Sheep Cervicovaginal Fluids; circles, TDF; squares, POC-TFV; triangles, TFV. (A) CVF Collected neat (Weck-Cel); TDF-FTC-EVG IVR group. (B) CVF Collected via lavage, corrected for dilution; TDF-FTC-EVG IVR group. (C) CVF Collected neat (Weck-Cel); TDF-FTC-EVG(Na) IVR group. (D) CVF Collected via lavage, corrected for dilution; TDF-FTC-EVG(Na) IVR group. Figure S4. Concentration-time Plots for TDF and its Metabolites in Macaque Cervicovaginal Fluids Collected Neat (Weck-Cel); circles, TDF; squares, POC-TFV; triangles, TFV. (A) CVF Collected proximal to IVRs; TDF-FTC-EVG IVR group. (B) CVF Collected distal to IVRs; TDF-FTC-EVG IVR group. (C) CVF Collected proximal to IVRs; TDF-FTC-EVG(Na) IVR group. (D) CVF Collected distal to IVRs; TDF-FTC-EVG(Na) IVR group.

## References

[1] Landovitz R.J., Donnell D., Clement M.E., Hanscom B., Cottle L., Coelho L., Cabello R., Chariyalertsak S., Dunne E.F., Frank I. (2021). Cabotegravir for HIV Prevention in Cisgender Men and Transgender Women. N. Engl. J. Med..

[2] Delany-Moretlwe S., Hughes J.P., Bock P., Ouma S.G., Hunidzarira P., Kalonji D., Kayange N., Makhema J., Mandima P., Mathew C. (2022). Cabotegravir for the Prevention of HIV-1 in Women: Results from HPTN 084, a Phase 3, Randomised Clinical Trial. Lancet.

[3] Bekker L.G., Das M., Abdool Karim Q., Ahmed K., Batting J., Brumskine W., Gill K., Harkoo I., Jaggernath M., Kigozi G. (2024). Twice-yearly Lenacapavir or Daily F/TAF for HIV Prevention in Cisgender Women. N. Engl. J. Med..

[4] Han K., D’Amico R., Sievers J., Brimhall D., Spears B., Taylor D., Dorey D., Benn P., Morgan L., Hareedy R. Phase I Study of Cabotegravir Long-acting Injectable Formulations Supports ≥4-Monthly Dose Interval. Proceedings of the 2024 Conference on Retroviruses and Opportunistic Infections (CROI).

[5] Jogiraju V., Pawar P., Yager J., Ling J., Shen G., Chiu A., Hughes E., Palaparthy R., Carter C., Singh R. (2025). Pharmacokinetics and Safety of Once-yearly Lenacapavir: A Phase 1, Open-label Study. Lancet.

[6] UNAIDS Fact Sheet 2025—Global HIV Satistics. https://www.unaids.org/sites/default/files/2025-07/2025_Global_HIV_Factsheet_en.pdf.

[7] Shapley-Quinn M.K., Manenzhe K.N., Agot K., Minnis A.M., van der Straten A. (2019). “We Are Not the Same”: African Women’s View of Multipurpose Prevention Products in the TRIO Clinical Study. Int. J. Womens Health.

[8] Schmidt H.A., Prochazka M., Ingold H., Reza-Paul S., Chidarikire T., Romyco I., Rodolph M. (2025). Seizing the Moment: The Potential of PrEP Choice and Innovation to Transform HIV Prevention. J. Int. AIDS Soc..

[9] van der Straten A., Shapley-Quinn M.K., Reddy K., Cheng H., Etima J., Woeber K., Musara P., Palanee-Phillips T., Baeten J.M., Montgomery E.T. (2017). Favoring “Peace of Mind”: A Qualitative Study of African Women’s HIV Prevention Product Formulation Preferences from the MTN-020/ASPIRE Trial. AIDS Patient Care STDS.

[10] Ridgeway K., Montgomery E.T., Smith K., Torjesen K., van der Straten A., Achilles S.L., Griffin J.B. (2022). Vaginal Rng Acceptability: A Systematic Review and Meta-analysis of Vaginal Ring Experiences from around the World. Contraception.

[11] Friedland B.A., Browne E.N., Roberts S.T., Ngure K., Nakalega R., Macdonald P., Mpongo C.N., Tenza S., Mhlanga N., Szydlo D. (2025). Higher Acceptability of the Monthly Dapivirine Ring Versus Daily Oral Pre-Exposure Prophylaxis Among Adolescent Girls and Young Women in Sub-Saharan Africa in the REACH Trial. J. Acquir. Immune Defic. Syndr..

[12] Moss J.A., Baum M.M., das Neves J., Sarmento B. (2014). Microbicide Vaginal Rings. Drug Delivery and Development of Anti-HIV Microbicides.

[13] Kruse W., Eggertkruse W., Rampmaier J., Runnebaum B., Weber E. (1991). Dosage Frequency and Drug Compliance Behavior—A Comparative Study on Compliance with a Medication to Be Taken Twice or 4 Times Daily. Eur. J. Clin. Pharmacol..

[14] Quraishi S., David A. (2000). Depot Haloperidol Decanoate for Schizophrenia. Cochrane Database Syst. Rev..

[15] Sershen S., West J. (2002). Implantable, Polymeric Systems for Modulated Drug Delivery. Adv. Drug Deliv. Rev..

[16] Kutilek V.D., Sheeter D.A., Elder J.H., Torbett B.E. (2003). Is Resistance Futile?. Curr. Drug Targets Infect. Disord..

[17] Bhanji N.H., Chouinard G., Margolese H.C. (2004). A Review of Compliance, Depot Intramuscular Antipsychotics and the New Long-acting Injectable Atypical Antipsychotic Risperidone in Schizophrenia. Eur. Neuropsychopharmacol..

[18] Haycox A. (2005). Pharmacoeconomics of Long-acting Risperidone: Results and Validity of Cost-effectiveness Models. Pharmacoeconomics.

[19] Small G., Dubois B. (2007). A Review of Compliance to Treatment in Alzheimer’s Disease: Potential Benefits of a Transdermal Patch. Curr. Med. Res. Opin..

[20] Yeaw J., Benner J.S., Walt J.G., Sian S., Smith D.B. (2009). Comparing Adherence and Persistence Across 6 Chronic Medication Classes. J. Manag. Care Pharm..

[21] Baeten J.M., Palanee-Phillips T., Brown E.R., Schwartz K., Soto-Torres L.E., Govender V., Mgodi N.M., Matovu Kiweewa F., Nair G., Mhlanga F. (2016). Use of a Vaginal Ring Containing Dapivirine for HIV-1 Prevention in Women. N. Engl. J. Med..

[22] Nel A., van Niekerk N., Kapiga S., Bekker L.G., Gama C., Gill K., Kamali A., Kotze P., Louw C., Mabude Z. (2016). Safety and Efficacy of a Dapivirine Vaginal Ring for HIV Prevention in Women. N. Engl. J. Med..

[23] Nel A., van Niekerk N., Van Baelen B., Malherbe M., Mans W., Carter A., Steytler J., van der Ryst E., Craig C., Louw C. (2021). Safety, Adherence, and HIV-1 Seroconversion among Women Using the Dapivirine Vaginal Ring (DREAM): An Open-label, Extension Study. Lancet HIV.

[24] Nair G., Celum C., Szydlo D., Brown E.R., Akello C.A., Nakalega R., Macdonald P., Milan G., Palanee-Phillips T., Reddy K. (2023). Adherence, Safety, and Choice of the Monthly Dapivirine Vaginal Ring or Oral Emtricitabine Plus Tenofovir Disoproxil Fumarate for HIV Pre-exposure Prophylaxis among African Adolescent Girls and Young Women: A Randomised, Open-label, Crossover Trial. Lancet HIV.

[25] Fonner V.A., Irungu E., Conlon M., Akello C.A., Gwavava E., K’Orimba K., Naidoo N.P., Jeckonia P., Mahaka I., Mullick S. (2025). PrEP Choice in the Real World: Results of a Prospective Cohort Study Describing Uptake and Use Patterns of Oral PrEP and the Dapivirine Vaginal Ring among Women in sub-Saharan Africa. J. Int. AIDS Soc..

[26] PrEPWatch The Dapivirine Vaginal Ring. https://www.prepwatch.org/products/dapivirine-vaginal-ring/.

[27] Baum M.M., Butkyavichene I., Gilman J., Kennedy S., Kopin E., Malone A.M., Nguyen C., Smith T.J., Friend D.R., Clark M.R. (2012). An Intravaginal Ring for the Simultaneous Delivery of Multiple Drugs. J. Pharm. Sci..

[28] Vincent K.L., Moss J.A., Marzinke M.A., Hendrix C.W., Anton P.A., Gunawardana M., Dawson L., Olive T.J., Pyles R.B., Baum M.M. (2018). Phase I Trial of Pod-intravaginal Rings Delivering Antiretroviral Agents for HIV-1 Prevention.: Rectal Drug Exposure from Vaginal Dosing with Tenofovir Disoproxil Fumarate, Emtricitabine, and Maraviroc. PLoS ONE.

[29] Vincent K.L., Moss J.A., Marzinke M.A., Hendrix C.W., Anton P.A., Pyles R.B., Guthrie K.M., Dawson L., Olive T.J., Butkyavichene I. (2018). Safety and Pharmacokinetics of Single, Dual, and Triple Antiretroviral Drug Formulations Delivered by Pod-intravaginal Rings Designed for HIV-1 Prevention: A Phase I Trial. PLoS Med..

[30] Moss J.A., Baum M.M., Malone A.M., Kennedy S., Kopin E., Nguyen C., Gilman J., Butkyavichene I., Willis R., Vincent K.L. (2012). Tenofovir and Tenofovir Disoproxil Pharmacokinetics from Intravaginal Rings. AIDS.

[31] Moss J.A., Srinivasan P., Smith T.J., Butkyavichene I., Lopez G., Brooks A.A., Martin A., Dinh C.T., Smith J.M., Baum M.M. (2014). Pharmacokinetics and Preliminary Safety Study of Pod-Intravaginal Rings Delivering Antiretroviral Combinations for HIV Prophylaxis in a Macaque Model. Antimicrob. Agents Chemother..

[32] Baum M.M., Butkyavichene I., Churchman S.A., Lopez G., Miller C.S., Smith T.J., Moss J.A. (2015). An Intravaginal Ring for the Sustained Delivery of Tenofovir Disoproxil Fumarate. Int. J. Pharm..

[33] Moss J.A., Butkyavichene I., Churchman S.A., Gunawardana M., Fanter R., Miller C.S., Yang F., Easley J.T., Marzinke M.A., Hendrix C.W. (2016). Combination Pod-intravaginal Ring Delivers Antiretroviral Agents for HIV Prophylaxis: Pharmacokinetic Evaluation in an Ovine Model. Antimicrob. Agents Chemother..

[34] Gunawardana M., Baum M.M., Smith T.J., Moss J.A. (2014). An Intravaginal Ring for the Sustained Delivery of Antibodies. J. Pharm. Sci..

[35] Zhao C., Gunawardana M., Villinger F., Baum M.M., Remedios-Chan M., Moench T.R., Zeitlin L., Whaley K.J., Bohorov O., Smith T.J. (2017). Pharmacokinetics and Preliminary Safety of Pod-Intravaginal Rings Delivering the Monoclonal Antibody VRC01-N for HIV Prophylaxis in a Macaque Model. Antimicrob. Agents Chemother..

[36] Gunawardana M., Mullen M., Yoo J., Webster P., Moss J.A., Baum M.M. (2014). Sustained Delivery of Commensal Bacteria from Pod-intravaginal Rings. Antimicrob. Agents Chemother..

[37] Srinivasan P., Moss J.A., Gunawardana M., Churchman S.A., Yang F., Dinh C.T., Mitchell J.M., Zhang J., Fanter R., Miller C.S. (2016). Topical Delivery of Tenofovir Disoproxil Fumarate and Emtricitabine from Pod-intravaginal Rings Protect Macaques from Multiple SHIV Exposures. PLoS ONE.

[38] Promadej-Lanier N., Smith J.M., Srinivasan P., McCoy C.F., Butera S., Woolfson A.D., Malcolm R.K., Otten R.A. (2009). Development and Evaluation of a Vaginal Ring Device for Sustained Delivery of HIV Microbicides to Non-human Primates. J. Med. Primatol..

[39] Moss J.A., Malone A.M., Smith T.J., Butkyavichene I., Cortez C., Gilman J., Kennedy S., Kopin E., Nguyen C., Sinha P. (2012). Safety and Pharmacokinetics of Intravaginal Rings Delivering Tenofovir in Pig-tailed Macaques. Antimicrob. Agents Chemother..

[40] Moss J.A., Malone A.M., Smith T.J., Kennedy S., Kopin E., Nguyen C., Gilman J., Butkyavichene I., Vincent K.L., Motamedi M. (2012). Simultaneous Delivery of Tenofovir and Acyclovir via an Intravaginal Ring. Antimicrob. Agents Chemother..

[41] National Research Council (2011). Guide for the Care and Use of Laboratory Animals.

[42] Smith J.M., Moss J.A., Srinivasan P., Butkyavichene I., Gunawardana M., Fanter R., Miller C.S., Sanchez D., Yang F., Ellis S. (2017). Novel Multipurpose Pod-intravaginal Ring for the Prevention of HIV, HSV, and Unintended Pregnancy: Pharmacokinetic Evaluation in a Macaque Model. PLoS ONE.

[43] Vincent K.L., Bell B.A., Rosenthal S.L., Stanberry L.R., Bourne N., Sweeney Y.T.C., Patton D.L., Motamedi M. (2008). Application of Optical Coherence Tomography for Monitoring Changes in Cervicovaginal Epithelial Morphology in Macaques: Potential for Assessment of Microbicide Safety. Sex. Transm. Dis..

[44] Vincent K.L., Bourne N., Bell B.A., Vargas G., Tan A., Cowan D., Stanberry L.R., Rosenthal S.L., Motamedi M. (2009). High Resolution Imaging of Epithelial Injury in the Sheep Cervicovaginal Tract: A Promising Model for Testing Safety of Candidate Microbicides. Sex. Transm. Dis..

[45] US FDA (2001). Guidance for Industry: Bioanalytical Method Validation.

[46] Churchman S.A., Moss J.A., Baum M.M. (2016). Accurate Measurement of Female Genital Tract Fluid Dilution in Cervicovaginal Lavage Samples. J. Chromatogr. B.

[47] Massud I., Nishiura K., Ruone S., Holder A., Dinh C., Lipscomb J., Mitchell J., Khalil G.M., Heneine W., Garcia-Lerma J.G. (2024). Weekly Oral Tenofovir Alafenamide Protects Macaques from Vaginal and Rectal Simian HIV Infection. Pharmaceutics.

[48] Kuklenyik Z., Martin A., Pau C.P., Holder A., Youngpairoj A.S., Zheng Q., Cong M.E., Garcia-Lerma J.G., Heneine W., Pirkle J.L. (2009). On-line Coupling of Anion Exchange and Ion-pair Chromatography for Measurement of Intracellular Triphosphate Metabolites of Reverse Transcriptase Inhibitors. J. Chromatogr. B.

[49] Lan P., Tonomura N., Shimizu A., Wang S., Yang Y.-G. (2006). Reconstitution of a Functional Human Immune System in Immunodeficient Mice through Combined Human Fetal Thymus/Liver and CD34^+^ Cell Transplantation. Blood.

[50] Melkus M.W., Estes J.D., Padgett-Thomas A., Gatlin J., Denton P.W., Othieno F.A., Wege A.K., Haase A.T., Garcia J.V. (2006). Humanized Mice Mount Specific Adaptive and Innate Immune Responses to EBV and TSST-1. Nat. Med..

[51] Sun Z.F., Denton P.W., Estes J.D., Othieno F.A., Wei B.L., Wege A.K., Melkus M.W., Padgett-Thomas A., Zupancic M., Haase A.T. (2007). Intrarectal Transmission, Systemic Infection, and CD4(+) T Cell Depletion in Humanized Mice Infected with HIV-1. J. Exp. Med..

[52] Stoddart C.A., Maidji E., Galkina S.A., Kosikova G., Rivera J.M., Moreno M.E., Sloan B., Joshi P., Long B.R. (2011). Superior Human Leukocyte Reconstitution and Susceptibility to Vaginal HIV Transmission in Humanized NOD-scid IL-2Rγ(−/−) (NSG) BLT Mice. Virology.

[53] Denton P.W., Nochi T., Lim A., Krisko J.F., Martinez-Torres F., Choudhary S.K., Wahl A., Olesen R., Zou W., Di Santo J.P. (2012). IL-2 Receptor Gamma-chain Molecule is Critical for Intestinal T-cell Reconstitution in Humanized Mice. Mucosal Immunol..

[54] Gallay P.A., Chatterji U., Kirchhoff A., Gandarilla A., Gunawardana M., Pyles R.B., Marzinke M.A., Moss J.A., Baum M.M. (2017). Prevention of Vaginal and Rectal HIV Transmission by Antiretroviral Combinations in Humanized Mice. PLoS ONE.

[55] Denton P.W., Estes J.D., Sun Z.F., Othieno F.A., Wei B.D.L., Wege A.K., Powell D.A., Payne D., Haase A.T., Garcia J.V. (2008). Antiretroviral Pre-exposure Prophylaxis Prevents Vaginal Transmission of HIV-1 in Humanized BLT Mice. PLoS Med..

[56] Denton P.W., Krisko J.F., Powell D.A., Mathias M., Kwak Y.T., Martinez-Torres F., Zou W., Payne D.A., Estes J.D., Garcia J.V. (2010). Systemic Administration of Antiretrovirals Prior to Exposure Prevents Rectal and Intravenous HIV-1 Transmission in Humanized BLT Mice. PLoS ONE.

[57] Denton P.W., Othieno F., Martinez-Torres F., Zou W., Krisko J.F., Fleming E., Zein S., Powell D.A., Wahl A., Kwak Y.T. (2011). One Percent Tenofovir Applied Topically to Humanized BLT Mice and Used According to the CAPRISA 004 Experimental Design Demonstrates Partial Protection from Vaginal HIV Infection, Validating the BLT Model for Evaluation of New Microbicide Candidates. J. Virol..

[58] Chateau M.L., Denton P.W., Swanson M.D., McGowan I., Garcia J.V. (2013). Rectal Transmission of Transmitted/Founder HIV-1 Is Efficiently Prevented by Topical 1% Tenofovir in BLT Humanized Mice. PLoS ONE.

[59] Gallay P.A., Chatterji U., Kirchhoff A., Gandarilla A., Pyles R.B., Baum M.M., Moss J.A. (2018). Protection Efficacy of C5A Against Vaginal and Rectal HIV Challenges in Humanized Mice. Open Virol. J..

[60] Baum M.M., Ramirez C.M., Moss J.A., Gunawardana M., Bobardt M., Gallay P.A. (2020). Highly Synergistic Drug Combination Prevents Vaginal HIV Infection in Humanized Mice. Sci. Rep..

[61] Gallay P.A., Ramirez C.M., Baum M.M. (2023). Acute Antagonism in Three-drug Combinations for Vaginal HIV Prevention in Humanized Mice. Sci. Rep..

[62] Chou T.C., Talalay P. (1984). Quantitative Analysis of Dose-effect Relationships: The Combined Effects of Multiple Drugs or Enzyme Inhibitors. Adv. Enzym. Regul..

[63] Chou T.C. (2006). Theoretical Basis, Experimental Design, and Computerized Simulation of Synergism and Antagonism in Drug Combination Studies. Pharmacol. Rev..

[64] Chou T.C., Martin N. (2005). CompuSyn for Drug Combinations: PC Software and User’s Guide: A Computer Program for Quantitation of Synergism and Antagonism in Drug Combinations, and the Determination of IC_50_ and ED_50_ and LD_50_ Values.

[65] Javanbakht M., Guerry S., Gorbach P.M., Stirland A., Chien M., Anton P., Kerndt P.R. (2010). Prevalence and Correlates of Heterosexual Anal Intercourse Among Clients Attending Public Sexually Transmitted Disease Clinics in Los Angeles County. Sex. Transm. Dis..

[66] Gorbach P.M., Pines H., Javanbakht M., Weiss R.E., Jeffries R., Cranston R.D., Fuchs E.J., Hezerah M., Brown S., Voskanian A. (2014). Order of Orifices: Sequence of Condom Use and Ejaculation by Orifice During Anal Intercourse Among Women: Implications for HIV Transmission. J. Acquir. Immune Defic. Syndr..

[67] Owen B.N., Brock P.M., Butler A.R., Pickles M., Brisson M., Baggaley R.F., Boily M.C. (2015). Prevalence and Frequency of Heterosexual Anal Intercourse Among Young People: A Systematic Review and Meta-analysis. AIDS Behav..

[68] Chou T.C. (2010). Drug Combination Studies and Their Synergy Quantification Using the Chou-Talalay Method. Cancer Res..

[69] Peet M.M., Agrahari V., Anderson S.M., Hanif H., Singh O.N., Thurman A.R., Doncel G.F., Clark M.R. (2019). Topical Inserts: A Versatile Delivery Form for HIV Prevention. Pharmaceutics.

[70] Dobard C.W., Peet M.M., Nishiura K., Holder A., Dinh C., Mitchell J., Khalil G., Pan Y., Singh O.N., McCormick T.J. (2022). Single Dose Topical inserts Containing Tenofovir Alafenamide Fumarate and Elvitegravir Provide Pre- and Post-exposure Protection against Vaginal SHIV Infection in Macaques. eBioMedicine.

[71] Makarova N., Singletary T., Peet M.M., Mitchell J., Holder A., Dinh C., Agrahari V., Mendoza M., Pan Y., Heneine W. (2022). Pharmacokinetics and Efficacy of Topical Inserts Containing Tenofovir Alafenamide Fumarate and Elvitegravir Administered Rectally in Macaques. eBioMedicine.

[72] Thurman A.R., Ouattara L.A., Yousefieh N., Anderson P.L., Bushman L.R., Fang X., Hanif H., Clark M., Singh O., Doncel G.F. (2023). A Phase I Study to Assess Safety, Pharmacokinetics, and Pharmacodynamics of a Vaginal Insert Containing Tenofovir Alafenamide and Elvitegravir. Front. Cell. Infect. Microbiol..

[73] O’Hanlon D.E., Come R.A., Moench T.R. (2019). Vaginal pH Measured in Vivo: Lactobacilli Determine pH and Lactic acid Concentration. BMC Microbiol..

[74] Miller E.A., Beasley D.E., Dunn R.R., Archie E.A. (2016). Lactobacilli Dominance and Vaginal pH: Why Is the Human Vaginal Microbiome Unique?. Front. Microbiol..

