# Supplementary material for: Preclinical Pharmacokinetics of a Triple-Combination Intravaginal Ring for HIV Prevention
Source: Pharmaceutics. 2026 Jul 7;18(7):829. doi: 10.3390/pharmaceutics18070829 (PMC13415328; doi:10.3390/pharmaceutics18070829)
Supplement: Supplementary file 1 [file pharmaceutics-18-00829-s001.zip › pharmaceutics-4330170-supplementary.pdf]

Supplementary Materials for:

Preclinical Pharmacokinetics of a Triple-combination Intravaginal Ring for HIV Prevention

John A. Moss*, Priya Srinivasan, Irina Butkyavichene, Manjula Gunawardana, Amalia E. Castonguay, John M. Cortez Jr., Patricia Galvan, Sofia Rivera, Jining Zhang, Chuong Dinh, Angela Holder, Dawn Little, Shanon Bachman, Kristen Kelley, Christina M. Ramirez, Philippe A. Gallay, Kathleen L. Vincent, James M. Smith, Marc M. Baum*

Summary:

Table S1

Table S2

Table S3

Fig. S1

Fig. S2

Fig. S3

Fig. S4

Table S1. Analytical limits of quantitation (LOQs) and ranges for drug and drug metabolites in sheep and macaque specimens. All values are reported in (ng $sample^{-1}$) unless otherwise specified. CVF, cervicovaginal fluid; CVL, cervicovaginal lavage; VT, vaginal tissue; RT, rectal tissue; RF, rectal fluid.

Sample Details		LOQ (ng $sample^{-1}$)	Range (ng $sample^{-1}$)
CVF			
	TDF	0.25[1]<br>9.0[2]	0.5 – 500[1]<br>0.5 – 1,000[2]
	POC-TFV	1.3[1]<br>33[2]	1 – 5,000[1]<br>1 – 10,000[2]
	TFV	20[1]<br>18[2]	5 – 25,000
	FTC	40[1]<br>10[2]	1 – 4,800[1]<br>1 – 15,000[2]
	EVG	1.4[1]<br>16[2]	1 – 15,000[1]<br>1 – 1,000[2]
CVL			
	TDF	5.0	0.5 – 100
	POC-TFV	1.0	0.5 – 2,500
	TFV	12	0.5 – 25,000
	FTC	10	1 – 5,000
	EVG	1.3	0.5 – 20,000
VT			
	TFV	1.0	0.5 – 20,00
	TFV-DP	100[3]	81 – 41,685
	FTC	1.0	0.5 – 2,000
	FTC-TP	500[3]	437 – 223,796
	EVG	1.0	0.5 – 2,000
RT			
	TFV	1.0	0.5 – 2,000
	TFV-DP	100[1]	81 – 41,685
	FTC	1.0	0.5 – 2,000
	FTC-TP	500[1]	437 – 223,796
	EVG	1.0	0.5 – 2,000
RF			
	TFV	1.0	1 – 93
	FTC	1.0	2 – 1,280
	EVG	1.0	52 – 2,950

[1] sheep samples; [2] macaque samples; [3] fmol $sample^{-1}$

Table S2. Colposcopic findings during TDF-FTC-EVG IVR study in sheep (*N* = 3).

Sheep ID	Day 0	Day 7	Day 14	Day 21	Day 28
P3	Pink, normal, petechiae (from speculum)	None	Mild erythema, intact vessels	Mottled, mild erythema, intact vessels	Mottled, mild erythema, intact vessels, diffuse vessels
P8	None	Intact vessels	Mild erythema, intact vessels	Mottled, mild erythema, intact vessels, diffuse vessels	Dark pink, mild erythema, intact vessels and diffuse vessels, no disruption
P57	None	Mild erythema, petechiae, intact vessels	Intact vessels	Mottled, mild erythema, petechiae, intact vessels, diffuse vessels	Dark pink, mottled, moderate erythema, petechiae, intact vessels, diffuse vessels, no disruption

Table S3. Colposcopic findings during TDF-FTC-EVG(Na) IVR study in sheep (*N* = 3).

Sheep ID	Day 0	Day 7	Day 14	Day 21	Day 28
P3	Intact vessels, diffuse vessels	Dark pink, mottled, mild erythema, intact and diffuse vessels, no disruption	Dark pink, mild to moderate erythema, intact and diffuse vessels, no disruption	Mild erythema, dark pink, intact and diffuse vessels, no disruption	Dark pink, mild erythema, petechiae, diffuse vessels, no disruption
P8	Pink, intact vessels, no disruption	Pink, mottled, mild erythema, intact vessels, diffuse vessels, no disruption	Mottled, moderate erythema, few petechiae, intact vessels, diffuse vessels, no disruption	Dark pink, intact vessels, diffuse vessels	Dark pink, severe erythema, petechiae, diffuse vessels, petechial hemorrhage, no disruption
P57	Mild erythema at healing biopsy sites only, no disruptions	Pink, normal to mild erythema, intact vessels, no disruption except old biopsy site	Dark pink, mottled, mild erythema, petechiae, intact and diffuse vessels	Dark pink, mild erythema, diffuse petechiae, diffuse vessels, no disruption	Dark pink, mild erythema, few petechiae, intact vessels, diffuse vessels

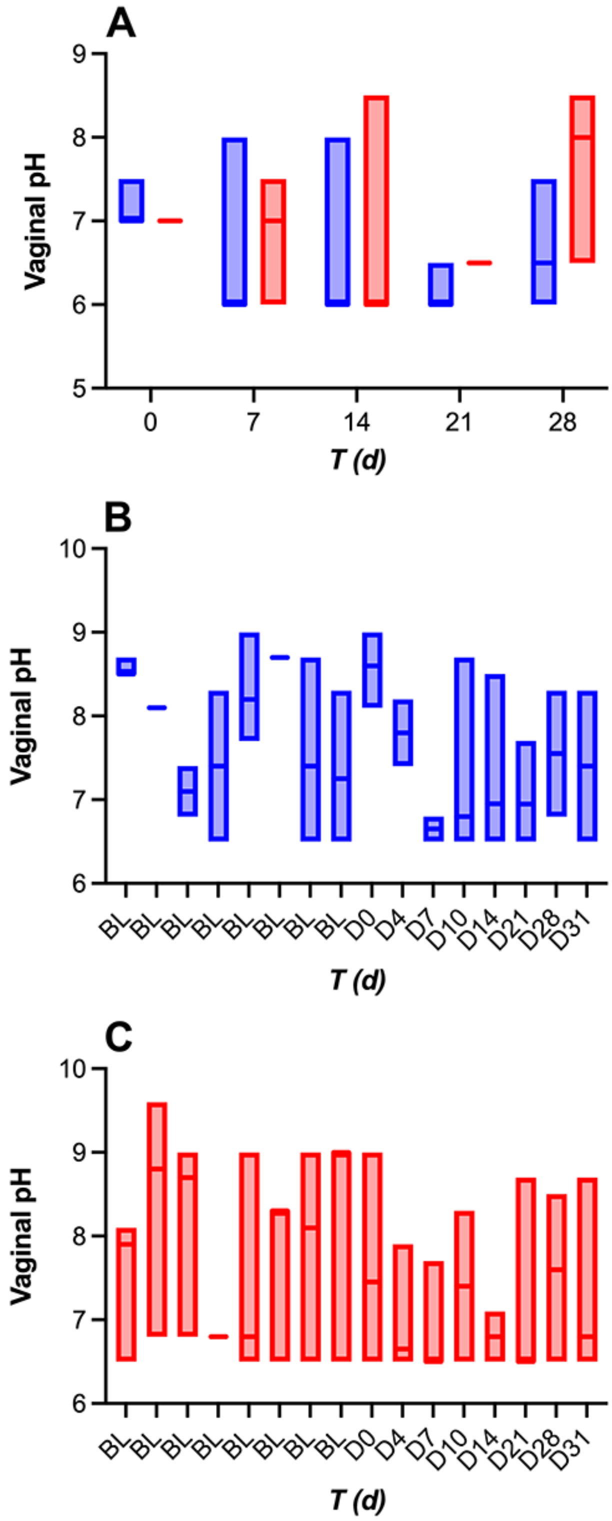

Figure S1. Temporal vaginal pH measurements. Box plots, line at median and floating bars represent minimum to maximum values. (**A**) Sheep; blue, TDF-FTC-EVG IVR group; red, TDF-FTC-EVG(Na) IVR group. (**B**) Macaques; TDF-FTC-EVG IVR group; (**C**) Macaques; TDF-FTC-EVG(Na) IVR group.

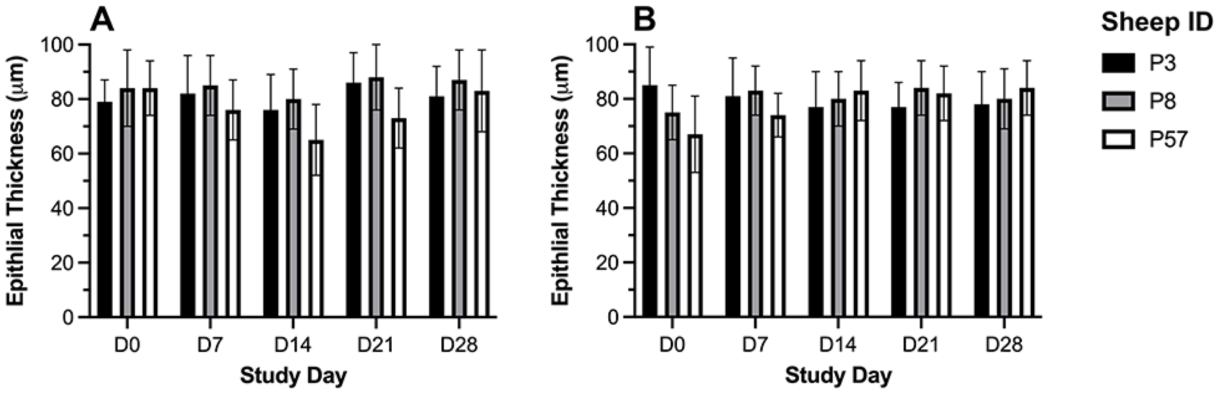

Figure S2. Temporal thickness variations in vaginal epithelium in sheep measured via optical coherence tomography (OCT). (**A**) TDF-FTC-EVG IVR group. (**B**) TDF-FTC-EVG(Na) IVR group.

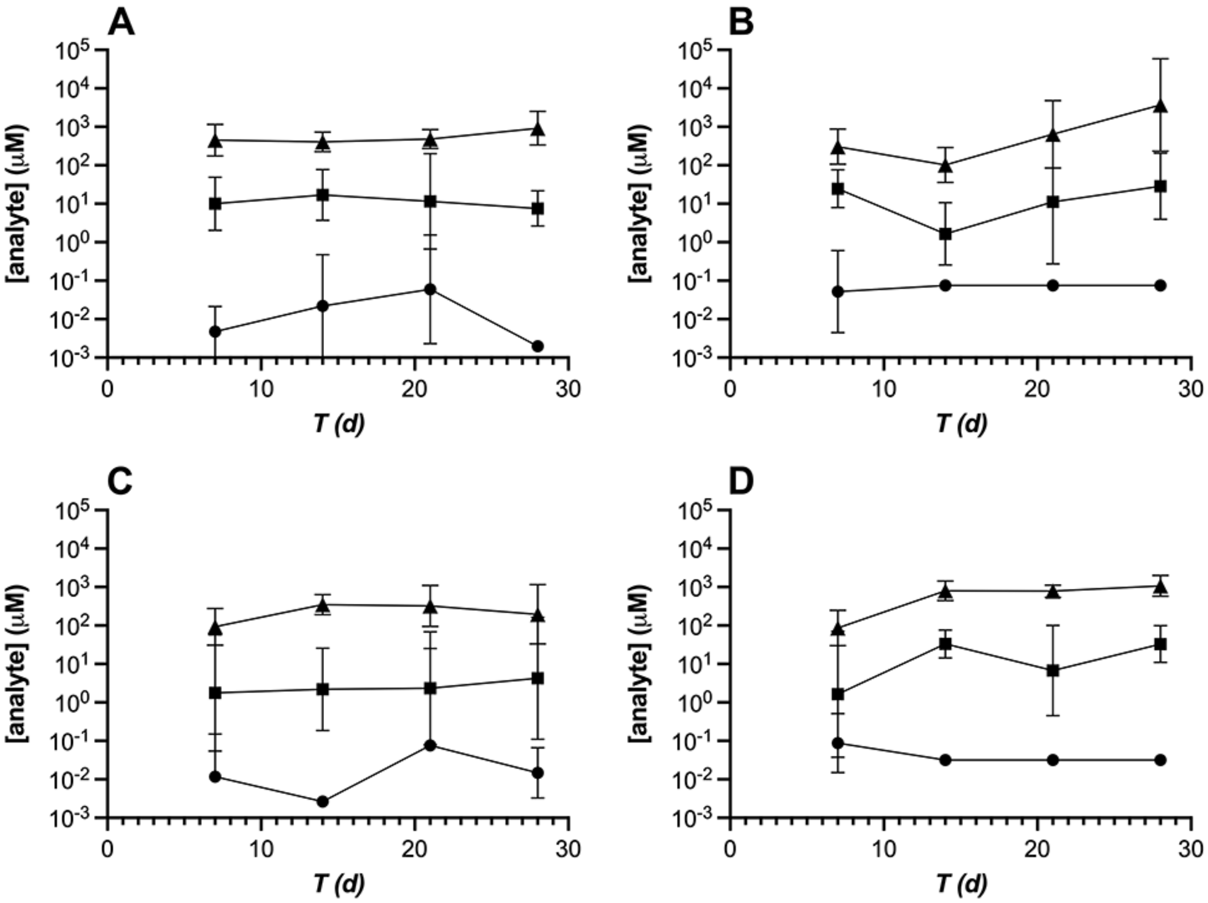

Figure S3. Concentration–time plots for TDF and its metabolites in sheep cervicovaginal fluids; circles, TDF; squares, POC-TFV; triangles, TFV. (**A**) CVF collected neat (Weck-Cel); TDF-FTC-EVG IVR group. (**B**) CVF collected via lavage, corrected for dilution; TDF-FTC-EVG IVR group. (**C**) CVF collected neat (Weck-Cel); TDF-FTC-EVG(Na) IVR group. (**D**) CVF collected via lavage, corrected for dilution; TDF-FTC-EVG(Na) IVR group.

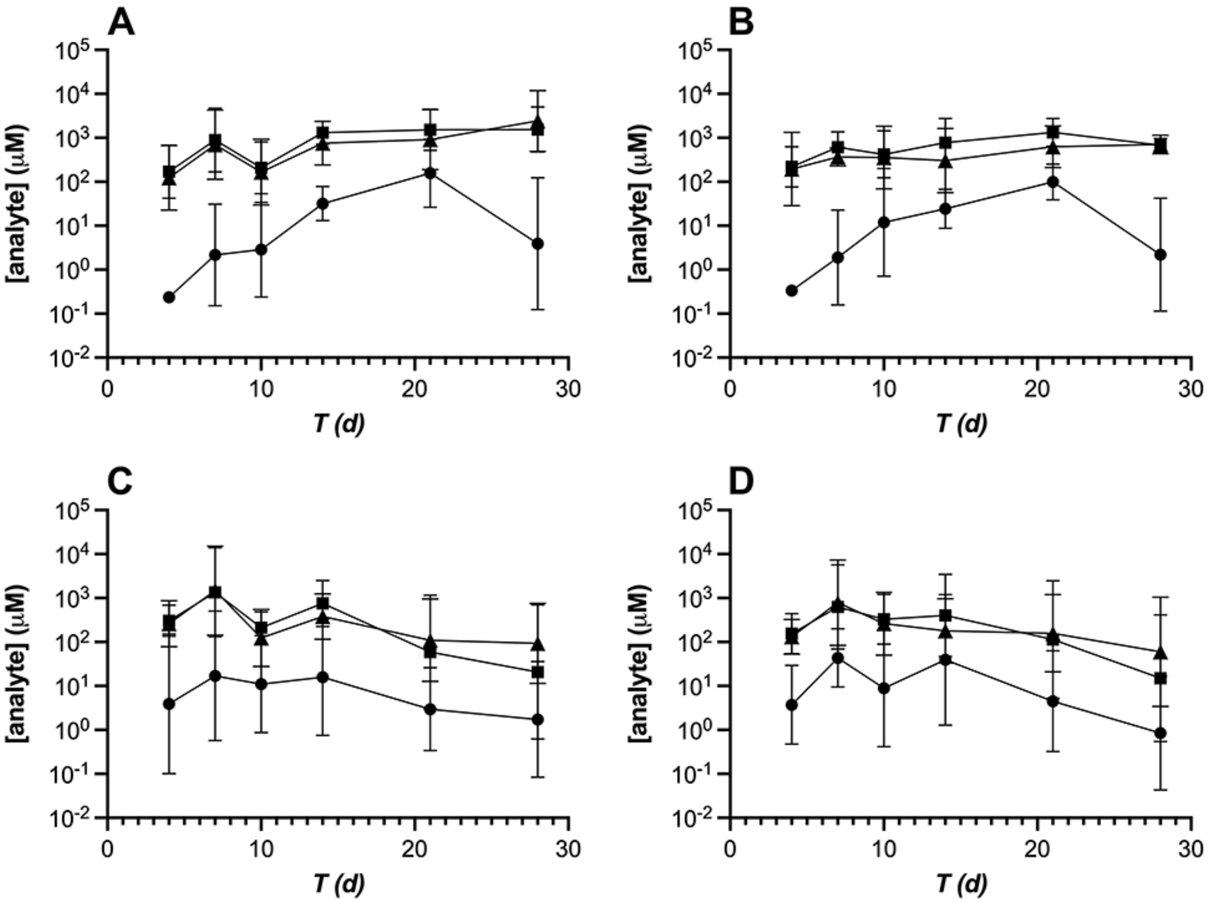

Figure S4. Concentration–time plots for TDF and its metabolites in macaque cervicovaginal fluids collected neat (Weck-Cel); circles, TDF; squares, POC-TFV; triangles, TFV. (**A**) CVF collected proximal to IVRs; TDF-FTC-EVG IVR group. (**B**) CVF collected distal to IVRs; TDF-FTC-EVG IVR group. (**C**) CVF collected proximal to IVRs; TDF-FTC-EVG(Na) IVR group. (**D**) CVF collected distal to IVRs; TDF-FTC-EVG(Na) IVR group.
